# Designing implementation strategies to improve identification, cascade testing, and management of families with familial hypercholesterolemia: An intervention mapping approach

**DOI:** 10.3389/frhs.2023.1104311

**Published:** 2023-04-28

**Authors:** Laney K. Jones, Evan M. Calvo, Gemme Campbell-Salome, Nicole L. Walters, Andrew Brangan, Gabriela Rodriguez, Catherine D. Ahmed, Kelly M. Morgan, Samuel S. Gidding, Marc S. Williams, Ross C. Brownson, Terry L. Seaton, Anne C. Goldberg, Mary P. McGowan, Alanna K. Rahm, Amy C. Sturm

**Affiliations:** ^1^ Department of Genomic Health, Research Institute, Geisinger, Danville, PA, United States; ^2^ Heart and Vascular Institute, Geisinger, Danville, PA, United States; ^3^ Geisinger Commonwealth School of Medicine, Geisinger College of Health Sciences, Geisinger, Scranton, PA, United States; ^4^ Department of Population Health Sciences, Research Institute, Geisinger, Danville, PA, United States; ^5^ Family Heart Foundation, Pasadena, CA, United States; ^6^ Prevention Research Center in St. Louis, Brown School, Washington University in St. Louis, St. Louis, MO, United States; ^7^ Department of Surgery (Division of Public Health Sciences), Alvin J. Siteman Cancer Center, Washington University School of Medicine, Washington University in St. Louis, St. Louis, MO, United States; ^8^ University of Health Sciences and Pharmacy in St. Louis, St. Louis, MO, United States; ^9^ Division of Endocrinology, Metabolism and Lipid Research, John T. Milliken Department of Internal Medicine, Washington University School of Medicine in St. Louis, Washington University in St. Louis, St. Louis, MO, United States; ^10^ 23andMe, Sunnyvale, CA, United States

**Keywords:** familial hypercholesterolemia, implementation science, intervention mapping, identification, cascade testing, treatment, management, cholesterol

## Abstract

**Introduction:**

Familial hypercholesterolemia (FH) is a common inherited cholesterol disorder that, without early intervention, leads to premature cardiovascular disease. Multilevel strategies that target all components of FH care including identification, cascade testing, and management are needed to address gaps that exist in FH care. We utilized intervention mapping, a systematic implementation science approach, to identify and match strategies to existing barriers and develop programs to improve FH care.

**Methods:**

Data were collected utilizing two methods: a scoping review of published literature, related to any component of FH care, and a parallel mixed method study using interviews and surveys. The scientific literature was searched using key words including “barriers” or “facilitators” and “familial hypercholesterolemia” from inception to December 1, 2021. The parallel mixed method study recruited individuals and families with FH to participate in either dyadic interviews (*N* = 11 dyads/22 individuals) or online surveys (*N *= 98 respondents). Data generated from the scoping review, dyadic interviews, and online surveys were used in the 6-step intervention mapping process. Steps 1–3 included a needs assessment, development of program outcomes and creation of evidence-based implementation strategies. Steps 4–6 included program development, implementation, and evaluation of implementation strategies.

**Results:**

In steps 1–3, a needs assessment found barriers to FH care included underdiagnosis of the condition which led to suboptimal management due to a myriad of determinants including knowledge gaps, negative attitudes, and risk misperceptions by individuals with FH and clinicians. Literature review highlighted barriers to FH care at the health system level, notably the relative lack of genetic testing resources and infrastructure needed to support FH diagnosis and treatment. Examples of strategies to overcome identified barriers included development of multidisciplinary care teams and educational programs. In steps 4–6, an NHLBI-funded study, the Collaborative Approach to Reach Everyone with FH (CARE-FH), deployed strategies that focused on improving identification of FH in primary care settings. The CARE-FH study is used as an example to describe program development, implementation, and evaluation techniques of implementation strategies.

**Conclusion:**

The development and deployment of evidence-based implementation strategies that address barriers to FH care are important next steps to improve identification, cascade testing, and management.

## 1. Introduction

Familial hypercholesterolemia (FH) is a common inherited cholesterol disorder (prevalence 1 in 250) which leads to premature cardiovascular disease when left untreated (1, 2). Patients with a pathogenic variant in an FH gene have triple the risk for atherosclerotic cardiovascular disease (ASCVD) when compared to those without a genetic variant at any low-density lipoprotein-cholesterol (LDL-C) level, due to lifelong exposure (3). Diagnosis is often made in middle-aged adults, after experiencing premature ASCVD (4). Event rates for an FH patient with prevalent ASCVD are 5-fold higher compared to those with no prior ASCVD (5). Treatment beginning in adolescence lowers the risk for ASCVD before age 40 years from about 25% to <1% (6, 7). Although diagnostic criteria and treatment guidelines exist, data from patient registries show that FH remains underdiagnosed and undertreated for decades (4, 8, 9).

Since FH is a disease that runs in families, it is imperative that family communication and cascade testing occur so that at-risk family members are notified of their risk and have the option to undergo testing for FH. Preliminary data from the MyCode Genomic Screening and Counseling Program at Geisinger showed that probands who received FH results had approximately three living at-risk first-degree relatives that should be notified of this diagnosis and their risk; however, cascade testing had only occurred for approximately 3.5% of those relatives. Strategies have been deployed in practice to address barriers for each component of FH care: identification, cascade testing, and management (10, 11). Such efforts include improving data monitoring, sending electronic notifications to clinicians, development of new clinical teams, etc. However, in the United States the identification gap has only been improved from 10% to 30% of people being diagnosed with FH and cascade testing efforts have been suboptimal (12, 13).

To date, a systematic implementation approach has not been taken to improve FH care. One method to systematically develop implementation strategies uses both intervention (14) and implementation mapping (15), and includes diverse stakeholder perspectives to inform and improve care (14, 16). The six-steps of intervention mapping build toward developing an intervention and its evaluation (14). The six-steps are: (1) needs assessment, (2) specifying change objectives, (3) selecting theory-based intervention methods and practical applications, (4) producing the program, (5) specifying implementation plans, and (6) generating an evaluation plan (14). Implementation mapping expands upon intervention mapping to add strategies to improve adoption, implementation, and maintenance. When a systematic approach has been applied in other health contexts, such as depression, there has been improvement in care (17, 18). In this paper, we describe a systematic adapted intervention (19) and implementation mapping approach, to identify and match implementation strategies to barriers to improve FH care.

## 2. Materials and methods

### 2.1. FH care

A comprehensive care approach for individuals and families with FH involves three components: identification of patients, cascade testing of at-risk family members, and effective lipid management of the affected individuals. Identification occurs when a patient meets clinical diagnostic criteria and/or has an identified disease-causing variant in one of the genes associated with FH. Cascade testing includes risk notification and testing of at-risk relatives for FH. Management is the clinical care path established by the clinicians and an individual patient with FH to reduce their cardiovascular event risk. Management is based on the application of evidence-based guidelines (1).

### 2.2. Data collection

Data on key determinants of FH care related to identification, cascade testing, and management including barriers and facilitators, attitudes, and perspectives were collected using two methods: (1) a scoping review of published literature, and (2) a mixed methods study using interviews and surveys.

#### 2.2.1. Scoping review

**Figure 1:**
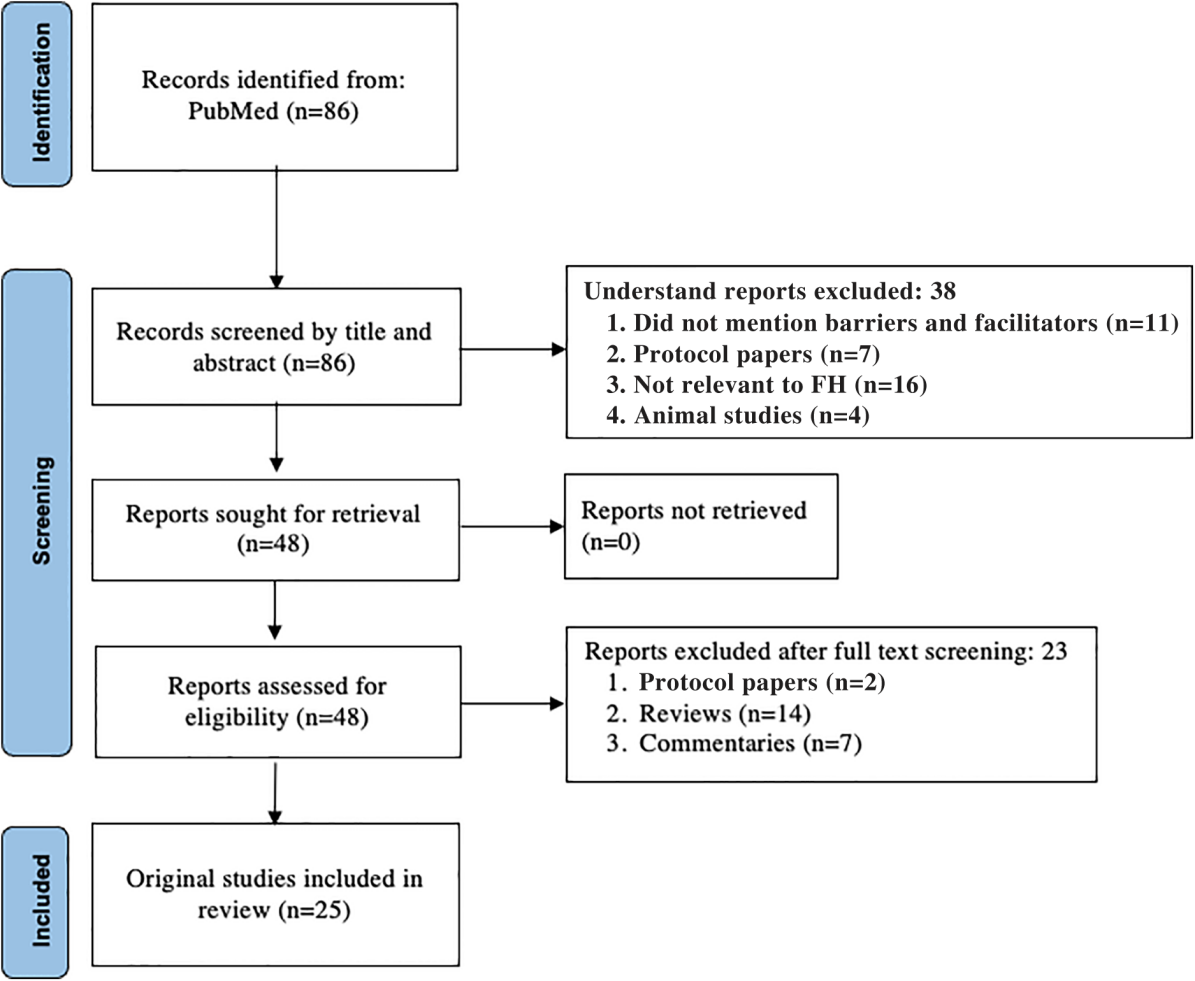
PRISMA diagram for scoping review.

A scoping review was performed to identify published literature related to any component of FH care. PubMed was searched using key words including “barriers” or “facilitators” and “familial hypercholesterolemia” from inception to December 1, 2021 (Table 1). Articles were excluded if they were not relevant to FH, not relevant to a component of FH care including identification, cascade testing, or management, or if the publication type was a narrative review, commentary, protocol-only, nonhuman, or were not published in the English language. This initial search resulted in a total of 86 potential articles; 25 articles were included in the analysis after the exclusion criteria were applied during abstract and full-text review (Figure 1). Articles were then categorized by component of FH care.

**Table 1:** PubMed search strategy for scoping review.

“barrier”[All Fields] OR “barriers”[All Fields] OR “facilitator”[All Fields] OR “facilitators”[All Fields] OR “enabler”[All Fields] OR “enablers”[All fields]	AND	“Hyperlipoproteinemia Type II”[Mesh] OR “familial hypercholesterolemia”[All Fields] OR “hyperlipoproteinemia type ii”[Mesh] OR (“hyperlipoproteinemia”[All Fields] AND “type”[All Fields] AND “ii”[All Fields]) OR “hyperlipoproteinemia type ii”[All Fields] OR (“familial”[All Fields] AND “hypercholesterolemia”[All Fields]) OR “familial hypercholesterolemia”[All Fields]

#### 2.2.2. Interviews and surveys

The mixed methods study recruited individuals and families with FH from Geisinger and the Family Heart Foundation to participate in either dyadic interviews (*n* = 11 dyads/22 individuals) or online surveys (*n* = 98 respondents). FH diagnosis was assigned by self-report or confirmed by genetic testing for those that participated in the MyCode Community Health Initiative at Geisinger (20). Two spouses participated in the dyadic interviews because they were the FH patient's caregiver and active in their care as well as communication with the family about FH. Dyadic phone interviews included the participant with an FH diagnosis and the family member they recruited to take part in the in-depth interview. Participants who completed interviews received a $20 Amazon gift card. Invitations to complete the online survey were sent *via* email to individuals identified through Geisinger and the Family Heart Foundation's databases, as well as *via* social media posts to the Family Heart Foundation's private groups. Snowball sampling was utilized by allowing survey respondents to invite their family members to complete a separate but similar version of the online survey. Survey participants were asked if they were the first person to be diagnosed with FH in their family. Survey respondents recruited from Geisinger were entered into a raffle to win one of five $50 Amazon gift cards. This recruitment strategy enabled us to have a sample of participants representing diverse diagnostic odysseys (i.e., journey to identification) and clinical experiences related to FH.

The interview guide and survey questions were developed through study team collaboration with the intent to elicit responses from participants about their experiences communicating about FH within their family (e.g., how did your family learn about FH? How does your family talk about FH?) (21, 22). Before interviews, participants were asked to review three family communication strategies and share their feedback on how to (re)design these strategies during the interviews (e.g., How can we improve the family letter? How would you want us to reach out to family members for a direct contact program?). Surveys were broken into three sections in which examples or additional information for each of the three family communication strategies was displayed and participants were asked open-ended questions about each strategy (e.g., How can we improve the family letter? How can we improve the chatbot? How would you want us to reach out to family members for a direct contact program?). The interview guide was tested with one dyad, iterated upon, and then deployed for all interviews thereafter (Supplementary File S1). Three separate versions of the survey were created for individuals with FH from Geisinger, individuals with FH from the Family Heart Foundation, and family members (Supplementary Files S2–S4). The combination of methods enabled triangulation of qualitative findings to capture the breadth and depth of experiences (23).

Audio-recorded dyadic phone interviews were transcribed, de-identified, and checked for accuracy before analysis. Open-ended survey responses were exported from the survey platform, de-identified, and checked for accuracy by ensuring there was only one IP address per response before inclusion in the full data set.

### 2.3. Procedures

This study deployed a modified version of intervention and implementation mapping (Table 2).

**Table 2:** Adapted intervention mapping steps for this study.

Step	Title	Description of activity	Data sources
1	Conduct a needs assessment	• Describe the problem of identifying and managing individuals with FH. • List factors which influence the identification, cascade testing, and management of individuals with FH. • Describe the target groups that influence FH care.	• Scoping review • Interviews/surveys
2	Program outcomes	• Define which behaviors and environmental conditions need to be changed to improve FH care • Describe who should make those changes and when. • Define the outcomes and make sure that they are specific, measurable, achievable, realistic, and designate a time frame to complete them.	
3	Theory and evidence-based strategies	• List barriers and facilitators which can be mapped to implementation strategies from existing evidence-based compilations	
4	Program development	• Develop an FH program that involves input from key stakeholders including persons with FH, family members, clinicians, health systems, researchers, advocacy organizations, and healthcare payers	• Published protocol paper
5	Implementation	• Develop an implementation plan. • Specify implementation outcomes of interest.	
6	Evaluation	• Develop an evaluation plan. • Decide which measurement tools exist to measure the program	

#### 2.3.1. Steps 1–3

Data generated from the scoping review, dyadic interviews, and online surveys were used to inform intervention mapping steps 1–3 (19). A scoping review was conducted to perform a needs assessment and uncovered reported barriers and facilitators to FH care in the literature for step 1. Two medical students and a senior researcher evaluated inclusion of the articles in abstract and full text screening in duplicate. Data from the scoping review was compared to conducted dyadic interviews (interviews with individuals with FH and their families) and online surveys to create a complete list of barriers and facilitators (step 1). Data from step 1 was used to develop program outcomes (step 2). In step 2, behaviors that promote better FH care were analyzed at the individual, clinician, and health system level. Determinants of those behaviors were extracted and ranked based on their changeability and importance. The ranking was performed by sending a survey to the study team that consists of FH researchers, advocates, and individuals with FH. Step 3 deviated from the original steps of intervention mapping, in that instead of selecting behavioral change interventions, implementation strategies were selected. This change occurred because evidence-based guidelines for FH care exist, and the purpose of the study was to improve guideline translation, so development of implementation strategies was necessary. Results from steps 1–2 were mapped to the Expert Recommendations for Implementing Change (ERIC), a compilation of evidence-based implementation strategies generated by implementation experts (step 3) (24). The ERIC compilation was chosen because the concepts from steps 1 and 2 more closely aligned with this list of strategies.

#### 2.3.2. Steps 4–6

To demonstrate steps 4–6 that include program development, implementation and evaluation, we provide an example of an FH program that was developed from the data generated in steps 1–3. This example includes a description of a funded study that is deploying implementation strategies to improve identification of FH in primary care.

## 3. Results

### 3.1. Demographics

#### 3.1.1. Data from the scoping review

A total of 25 studies were included from a scoping review of the literature (Table 3). These studies were published between 2002 and 2021 and mention at least one component of FH care: 12 referenced identification, 8 referenced cascade testing, and 16 referenced management with several addressing more than one component. Only two studies addressed all three components of care (26, 45). Most studies reported on barriers and facilitators to FH care.

**Table 3:** Original research studies identified from the scoping review of the literature.

Study	Year published	FH care category			Study population	Barriers (*n* = 25)	Facilitators (*n* = 15)
		Identification (*n* = 12)	Cascade testing (*n* = 8)	Management (*n* = 16)			
Baldry, E. et al. (25)	2021		X		Adults with FH	X	X
Block, R. C. et al. (26)	2021	X	X	X	Clinicians	X	
Mszar, R. et al. (27)	2021	X		X	Adults with FH	X	X
Soukup, J. et al. (28)	2021	X			Clinicians	X	X
Wong, N.D. et al. (29)	2021			X	Clinicians	X	
Allen-Tice, C. et al. (30)	2020	X			Children with FH	X	
Gidding, S. S. et al. (31)	2020	X	X		Adults with FH	X	X
Jackson, C. L. et al. (32)	2020	X		X	Adults with FH	X	
Jones, L. K. et al. (10)	2020			X	Adults with FH & Clinicians	X	X
Kinnear, F. J. et al. (33)	2020			X	Adults with FH	X	X
McCormick, D. et al. (34)	2020			X	Clinicians & Payers	X	
Unim, B. et al. (35)	2020	X			Clinicians	X	X
Kinnear, F. J. et al. (11)	2019			X	Adults with FH	X	X
Zimmerman, J. et al. (36)	2019	X			Clinicians	X	X
Yamashita, S. et al. (37)	2019			X	Clinicians	X	
Farwati, M. et al. (38)	2018	X		X	Adults with FH & Clinicians	X	X
van El, C. G. et al. (39)	2018		X		Clinicians & other stakeholders	X	X
Wurtmann, E. et al. (40)	2018		X		Parents of children with FH	X	X
Zafrir, B. et al. (41)	2018	** **		X	Adults with FH	X	X
Campbell, M. et al. (42)	2017	X	X		Adults with FH	X	
Cohen, J. D. et al. (43)	2017			X	Clinicians	X	X
Benson, G. et al. (44)	2016		X	X	Women with FH	X	
Hardcastle, S. J. et al. (45)	2015	X	X	X	Adults with FH	X	X
Frich, J. C. et al. (46)	2006	X		X	Women with FH	X	
Whayne, T. F. et al. (47)	2002			X	Patients eligible for lipid apheresis	X	

#### 3.1.2. Data from interviews and surveys

A total of 120 participants completed a dyadic interview or survey. Eleven family dyads (*n* = 22 individuals) were interviewed between July and August 2020. Detailed demographic information is available in Table 4.

**Table 4:** Demographics of interview and survey participants.

Total Participants (*N* = 120)	*n*	(%)
**Sex**		
Female	90	(75)
Male	30	(25)
**FH Diagnosis/Risk Status**		
Diagnosed	109	(90.8)
At-risk	6	(5)
Not at risk (spouse/caregiver)	5	(4.2)
**Educational Attainment**		
Some high school/high school diploma/GED	17	(14.2)
Some college or trade/technical degree	19	(15.8)
Associate's degree	8	(6.7)
Bachelor's degree	42	(35)
Post-graduate work or degree	33	(27.5)
Preferred not to answer	1	(0.8)
Dyadic Interview Participants (*n *= 22 individuals/11 dyads)	***n* individuals, *n* dyads**	**(%)**
**Dyad Type**		
Sisters	6, 3	(27.2)
Mother-Daughter	6, 3	(27.2)
Father-Daughter	2, 1	(9.1)
Mother-Son	4, 2	(18.2)
Spouses	4, 2	(18.2)
Survey Respondents (*n *= 98)	***n***	**(%)**
**Respondent Type**		
Individual with FH from Geisinger	19	(19.4)
Individual with FH from the Family Heart Foundation	72	(73.2)
Family member of an individual with FH	7	(7.1)

### 3.2. Step 1: needs assessment

#### 3.2.1. Summary of implementation problems identified

Data extracted from the scoping review of the literature, interviews, and surveys illuminated the barriers related to caring for individuals with FH and their families. These challenges can be categorized into three areas: identification, cascade testing, and management. Identification of FH has been a known problem worldwide with only 10%–30% of individuals estimated to have been diagnosed with FH (12, 13). Under-identification of FH has resulted in part from lack of a universally accepted definition for FH. To date, FH can be diagnosed *via* the presence of clinical criteria such as, high cholesterol levels and presence of family history with or without physical exam features. Multiple clinical screening tools exist, but there is not a gold standard. Alternatively, individuals can have a genetic diagnosis of FH by having a disease-causing variant in one of the genes associated with FH. There are also biological differences on how the ultimate health outcome, cardiovascular disease, presents in men as compared to women. Age-related differences in cholesterol levels exist due to the presence of other factors that affect lipid values over time, including environmental factors and diet. Limited screening during childhood has made it more difficult to prevent premature heart disease in early adulthood. Although, lipid screening in children is more discriminatory because children have not developed other risk factors thus if a high level of LDL-C is detected it is more likely to represent FH. By screening in childhood, it is also more likely to find an undiagnosed parent. Limitations due to privacy, family dynamics, geography, and other health and non-health-related concerns have presented family communication and cascade testing challenges. After identification and diagnosis, individuals with FH often receive suboptimal treatment. Women and children are less likely to be treated than adult men (48). Management of FH often requires daily combination lipid lowering therapy for life which can make adherence difficult.

#### 3.2.2. Barriers and facilitators influencing behaviors and environmental conditions

Barriers and facilitators were categorized into three levels: individual-, clinician-, and health system-level (Table 5).

**Table 5:** Description of behaviors influencing FH care identified through published and unpublished literature.

	Identification	Cascade testing	Management
**Individual level**			
Lack of awareness	X (10, 33)*		X (33, 38)*
Cost	X (31)*	X (31, 39)*	X (29, 47)*
Insurance coverage (absence of or limited coverage)	X (38)*		X (29, 34, 38, 41)*
Non-adherence			X (33, 38, 41, 44, 45)*
Side effects			X (41, 43, 44)*
Competing family demands		X (33, 45)	X (10, 33)
Competing personal demands		X (33, 44)	X (10, 33)
Stigma & health anxiety	X (27, 45)	X (45)	
Familial communication and social dynamics	X (27, 45)	X (31, 33, 40, 42, 44, 45)*	X (11)*
Privacy concerns & discrimination	X (27, 31)	X (39)	
Not achieving goal LDL-C levels with current therapies			X (43, 47)
Access to healthcare	X (27, 31)		X (27)
Access to patient support organizations	X (40)	X (40)	
Positive relationships with and attitudes towards physicians and healthcare system	X (27)*		X (29)*
Legal concerns	X (42)	X (42)	
**Clinician level**			
Lack of awareness	X (10, 26, 31, 32, 35, 36, 38)	X (31)	X (26, 30, 37, 38)
Belief that there is a lack of evidence	X (10)		X (32, 34)
Perception	X (10)		X (10)
Other clinical demands	X (28, 36)	X (39)	X (10, 29)
Inadequate record keeping systems		X (32, 36)	X (32)
Insurance (poor reimbursement for FH screening, time consuming PA procedures)	X (36, 38)		X (38, 43)
Skill level and comfort with genetic disorders	X (28, 36, 38)		
Education	X (28, 35)		X (11)
Lack of awareness of women's health needs			X (46)
**Health system level**			
Gaps in access to care			X (10)
Genetic testing resources and associated support staff/infrastructure	X (28, 31, 35, 36)	X (10, 39, 40)	X (11, 38)
Lack of formal screening programs that emphasize shared decision making			X (27)

Number denotes published article reference.

* Denotes from surveys and interviews generated by study team.

##### 3.2.2.1. Individual level

Barriers to FH identification, cascade testing, and management at the individual level include a lack of awareness of FH, which limits patients' ability to access testing and treatments. Additionally, interview and survey participants described ambivalent attitudes as another potential barrier to FH testing and treatments. Specifically, participants discussed how they or family members believed FH was not a serious condition or diagnosis, was not distinct from elevated cholesterol due to lifestyle, and the sense that high cholesterol is “the norm” in their family and to be expected. Participants described these attitudes as potentially undermining medical information about FH and as reducing likelihood they or their family members would feel a need to identify their high cholesterol FH or change current health management for high cholesterol. Next, while the cost of genetic testing is generally decreasing, financial concerns are still cited as a barrier to patient identification and family cascade testing, including confusion around the availability of insurance coverage for all types of testing for FH. Treatment costs and insurance coverage concerns also represent barriers when it comes to treatment(47), particularly with newer (brand-only) FDA-approved treatments, such as the proprotein convertase subtilisin/kexin type 9 (PCSK9) monoclonal antibodies and use of procedures such as low-density lipoprotein (LDL) apheresis. Treatment-related side effects, especially those attributed to statins, were also reported as a barrier to FH management. Monotherapy with statins is often unable to provide sufficient LDL cholesterol (LDL-C) lowering and has been cited as a barrier in the management of FH.

Competing personal and family demands may also prevent individuals with FH from communicating with their families about cascade testing and preventing them from prioritizing their health (e.g., adhering to treatments and lifestyle modifications). Similarly, some individuals with FH report difficulty contacting family members for cascade testing due to social and intra-familial communication dynamics (e.g., patients who no longer communicate with some or all family members). Individuals with FH and their families report a fear of a loss of privacy of their genetic information if they were to be tested, or discrimination from insurance companies if they were to receive a genetic diagnosis of FH. While there are laws that protect health information and prohibit the use of genetic information by health insurers and most employers, many individuals are unaware of such protections or do not trust that these laws will protect their information.

Finally, it has also been noted that stigma and health anxiety may prevent FH patients and their families from getting tested (i.e., FH patients do not want to be diagnosed with a serious medical condition). Limited access to healthcare and lack of patient support groups have also been reported as factors that may impede FH identification, cascade testing, and management.

Participants described experiences in which clinicians gave incorrect information such as suggesting that high LDL-C levels were acceptable without further treatment options, cascade testing was not necessary for at-risk relatives, or exhibiting poor interpersonal interactions to scare individuals about their FH-related health risks. These experiences created a barrier related to trust in clinicians and the healthcare system that participants explained complicated their ability to undergo testing for FH and/or receive appropriate treatment recommendations.

##### 3.2.2.2. Clinician level

At the clinician level, lack of awareness of FH as a specific genetic condition has also been cited as a barrier to testing in the index case, cascade testing, and management. Some clinicians believe that there is a lack of evidence to support FH identification and treatment, and a lack of FH-related education has been cited as a barrier to FH identification, cascade testing, and management among clinicians. Some clinicians (e.g., primary care clinicians) may also not feel comfortable with identifying genetic disorders in general, which hampers FH index patient identification. Clinicians may also feel that competing clinical demands (e.g., other health issues they need to cover with patients in short visits), affect their ability to initiate FH identification, cascade testing, and management. Inadequate record keeping systems also impede clinicians' ability to detect index cases with FH and initiate cascade testing. Clinicians also cite a lack of reimbursement as a limiting factor in FH identification and treatment. Finally, cascade testing is seen as difficult by some due to clinicians' legal concerns about making direct contact with a proband's family members.

##### 3.2.2.3. Health system level

At the health system level, access to care, particularly access to specialists, has been cited as a barrier to FH management. Similarly, organized FH screening programs that would facilitate FH identification and cascade testing are lacking. Healthcare systems, in general, may not have the infrastructure and resources (e.g., lipid-management specialists, genetic counseling programs, etc.) necessary to meet the needs of the FH population.

#### 3.2.3. Step 2: program outcomes and objectives

##### 3.2.3.1. Behaviors that promote better FH care

###### 3.2.3.1.1. Individual level

The level of individual patient and family knowledge of FH and its genetic basis potentially impacts FH identification. Individuals with FH felt that learning of their condition, and its specific genetic basis, is important and will prompt them to communicate the result with their at-risk relatives. They felt this would likewise prompt their relatives to undergo testing for FH and improve both their and their relatives' adherence to management recommendations. In addition, individuals can encourage screening when discussing the FH result with their at-risk relatives. Individuals who understand the importance of taking their medications as prescribed are often more willing to discuss medication-related side effects with their clinicians.

###### 3.2.3.1.2. Clinician level

Behaviors that affect identification include knowledge and implementation of guideline recommendations to screen for and identify FH. It is important for clinicians to understand that earlier identification is key to preventing future cardiovascular disease. Clinicians can also promote and facilitate communication between the individual with FH and their at-risk relatives. Clinicians have a key role in understanding and recommending appropriate treatment options and intensify treatment regimens.

###### 3.2.3.1.3. Health system level

Health systems may help to improve components of FH care by implementing protocols that make screening for FH easy for both the initial patient identified and their at-risk relatives, allow clinicians including primary care clinicians, specialists, and other healthcare clinicians to share responsibilities for FH care and remove barriers to ordering of testing and medications for FH. A health system's central laboratory could adopt language on lipid results prompting the clinician to consider FH when an LDL-C is found to be over 190 mg/dl.

##### 3.2.3.5. Determinants

Based on the barriers and facilitators identified through the mixed methods study, determinants are ranked by their ability to be addressed (changeable) and their contribution to the behavior (important) at the individual, clinician, and health system level (Table 6). The most mentioned determinants across all levels of care were knowledge, attitude, and risk perception. These determinants will serve as priority topics for development of implementation outcomes.

**Table 6:** Description of determinants identified for FH care.

Determinants	Important	Changeable
**Patient level**		
Knowledge	++	++
Attitude	++	+
Risk perception	++	++
Healthcare insecurity	+	+
Cost uncertainty	+	+
Self-efficacy	++	+
Social norms	+	+
**Clinician level**		
Knowledge	++	++
Risk perception	++	++
Skills	+	++
Attitude	++	+
Social norms	+	0
Cost uncertainty	+	+
Self-efficacy	+	++
Time	+	0
**Health system level**		
Value	++	++
Return on investment	++	+
Resources/processes/infrastructure	++	+
Time	+	0

Important: contribute significantly to the behavior (0, +, ++).

Changeable: ability to be changed (0, +, ++).

Co-authors that include FH experts, FH researchers, FH clinicians, and individuals with FH reviewed the determinants and ranked their importance and changeability based on their expertise and experiences.

#### 3.2.4. Step 3: implementation strategies mapped to program outcomes and objectives

Implementation strategies that can be deployed to help translate the evidence into clinical practice are detailed in Table 7. These implementation strategies were mapped to program outcomes and objectives and were standardized using the ERIC compilation. These strategies may provide a guide that can be used to develop tailored implementation strategies for a specific component of FH care. Most of these implementation strategies can be defined to address each component of FH care.

**Table 7:** Examples of implementation strategies that address determinants that relate to all components of FH care.

Determinant addressed	Level(s)	ERIC compilation implementation strategies	Definition*
Value	Health system	Alter financial incentives	Change patient cost, reimbursement fees, or other costs associated with uptake of the implementation
Resources, process, and infrastructure	Health system	Change accreditation, membership, or credentialing requirements	Change the requirements for accreditation or membership
Resources, process, and infrastructure	Health system	Change liability laws	Propose policy changes that would make implementing FH care easier
Resources, process, and infrastructure, time	Health system	Change record systems	Implement record systems to understand the impact of the implementation
Knowledge, risk perception, skills, attitude, self-efficacy, cost uncertainty	Clinician	Conduct dynamic educational meetings and ongoing training	Conduct initial and ongoing educational meetings or trainings on the implementation that are applicable to multiple learning styles
Value, Resources, process, infrastructure	Health system	Create a learning collaborative	Facilitate the formation of a group of FH clinicians or health systems focused on improving FH care
Resources, process, and infrastructure, time	Health system	Create new clinical teams or revise professional roles	Added different disciplines and skills sets or changes roles to improve uptake of the implementation
Knowledge, attitude, risk perception, skills, social norms	Clinician	Develop and distribute educational material	Develop and distribute information on how to implement better FH care
Resources, process, and infrastructure	Health system	Facilitate relay of clinical data to clinicians	Provide up-to-date data on the uptake of the implementation to clinicians
Knowledge, attitude, risk perception, skills, social norms, value	Health system, clinician, patient	Identify and prepare champions	Identify and prepare FH champions who support, market, and prompt the implementation overing barriers in the health system
Knowledge, attitude, risk perception, healthcare insecurity, cost uncertainty, self-efficacy, social norms	Patient	Intervene and involve patients and family members to enhance uptake and adherence	Engage patients and family members in the implement of FH care
Value	Health system	Mandate change	Leadership declares the implementation a priority
Knowledge, attitude, risk perception, skills, social norms	Health system, clinician	Promote network weaving	Identify and build relationships within and outside the health system to promote information sharing and collaborative problem-solving
Value, Resources, process, and infrastructure, time, return on investment	Health system	Provide ongoing consultation	Provide access to implementers to ensure smooth implementation
Resources, process, and infrastructure	Health system	Remind clinicians	Develop systems to remind clinicians to use the implementation
Knowledge, attitude, risk perception, social norms	Patient, clinician, health system	Use mass media	Use media to reach many patients, clinicians or health systems about FH care
Knowledge, attitude, risk perception, skills, social norms, value, time	Health system, clinician	Use train-the-trainer strategies	Train clinicians and health systems to deliver the implementation to others

ERIC, Expert Recommendation for Implementing Change.

* Definitions are adapted by the ERIC compilation definitions for FH care.

To ensure success and sustainability after deployment of the implementation strategies, it is important to develop a plan to obtain ongoing feedback from all stakeholders involved, reexamine the implementation outcomes, and provide assistance at the level of the patient, family, and clinician up to the health system, to help improve utility of the implementation strategies. These strategies will provide information on whether the implementation should be altered based on external and internal factors.

### 3.3. Example of a current FH program with an implementation and evaluation plan

Steps 4–6 were satisfied by designing CARE-FH (Collaborative Approach to Reach Everyone with FH), a clinical trial of implementation strategies, is funded by National Heart Lung and Blood Institute, and based on findings from steps 1–3.The goal of CARE-FH is to improve FH identification in primary care (49).

#### 3.3.1. Step 4: design the intervention

**Figure 2:**
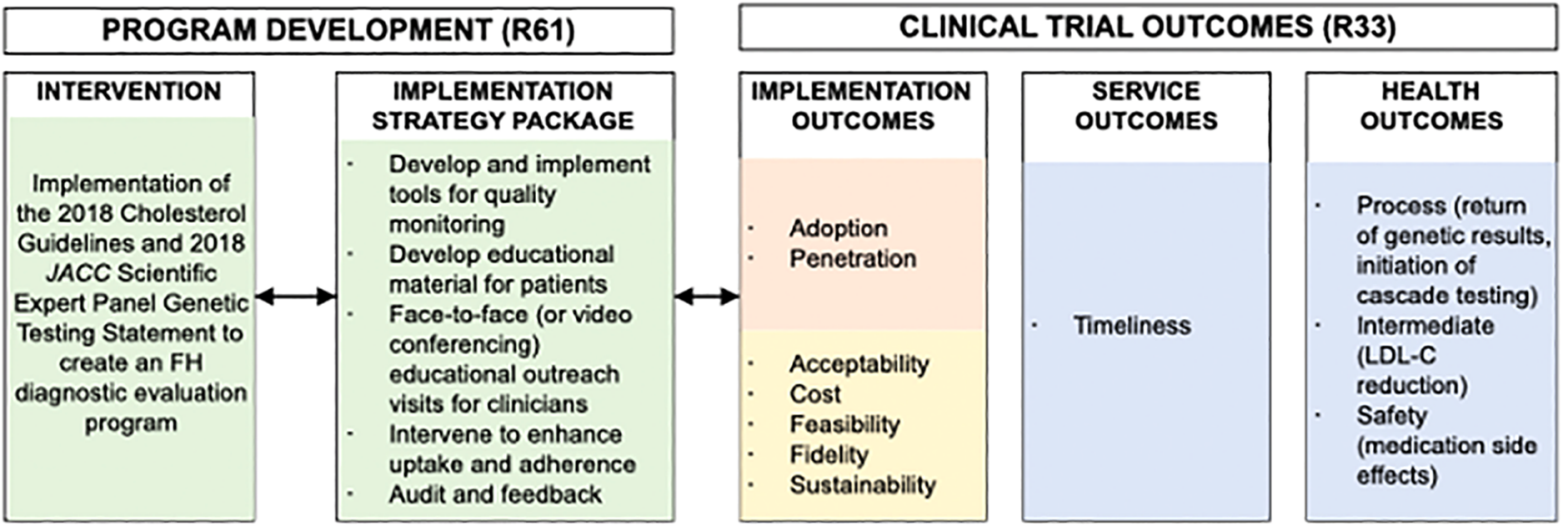
Adapted conceptual model of implementation research for the CARE-FH study. Reproduced under the CC-BY license from Jones LK et al. (49).

Steps 1–3 determined that there was a gap in translating evidence-based guidelines related to screening for FH into practice. The needs assessment found that screening for FH is recommended but not routinely performed. The decision was made that the evidence-based guidance would be based on the 2018 AHA/ACC/Multi-society Cholesterol Guidelines and the 2020 Expert Consensus Genetic Testing Statement (1, 50). These evidence-based guidelines were used to generate the diagnostic screening algorithm for clinicians to screen for FH in the study (Figure 2).

#### 3.3.2. Step 5: create an implementation approach

The CARE-FH study team selected implementation strategies relevant to FH identification from Table 7. These implementation strategies included: conduct dynamic educational meetings and ongoing training, develop and distribute educational material, intervene and involve patients and family members to enhance uptake and adherence, remind clinicians, and facilitate relay of clinical data to clinicians. The proposed comprehensive multi-level implementation strategy package was based on the ERIC compilation (Table 8). These implementation strategies were tailored to the Geisinger primary care practice using a 1-year pre-implementation phase where surveys, contextual inquiries, and pilot testing of the strategies was conducted.

**Table 8:** CARE-FH study proposed multi-level implementation strategies. Reproduced under the CC-BY license from Jones LK et al. (49).

Name of strategy*	Specific study definition	Actor	Action	Action target
Develop and implement tools for quality monitoring	EHR tools to order labs, record results, and document FH care	ImpT, MedT, and InfT	Use EHR to record, order, and prescribe FH Care	Service and health outcomes
Develop educational materials	Education regarding guidelines for identification and treatment of FH	MedT and InfT	Create a CME course for clinicians about FH. Explore clinician workflow and educational needs to design novel focused educational interventions integrated within clinical workflows to support evidence-based care	MedT ready to train clinicians on FH
Conduct educational outreach visits	CME educational material for FH that is presented to each clinic	MedT and clinicians	Attend CME course on FH	Improve knowledge about FH
Intervene with patients to enhance uptake and adherence	Reach out directly to patients to recommend screening for FH	Clinicians and ImpT	Letter sent to the patient. Clinician schedules patient for appointment.	Patients diagnosed with FH from those at-risk
Identify and prepare champions	Clinical lipid champions	MedT	Identify and train lipid champions	Improved performance of study metrics, reduced costs
Stage FH care delivery model scale up	Develop the timeline for the stepped-wedge rollout to primary care	Leadership team	Notify practices of roll out and schedule education	Begin the trial
Audit and provide feedback	Provide aggregate level feedback to clinics on diagnosing FH	MedT, InfT, and clinical leadership	Report back to clinicians’ aggregate level data	Improve effectiveness of the FH Diagnosis Program
Advisory board review	Clinical trial protocol	Advisory Board	Provide feedback on the clinical trial regarding protocol, generalizability and ethical issues	Protocol revision based on feedback

EHR, electronic health record; CME, continuing medical education; FH, familial hypercholesterolemia; ImpT, implementation science team; InfT, informatics and data science team; MedT, medical science team.

* Mapped to the Expert Recommendations for Implementing Change (ERIC) compilation.

#### 3.3.3. Step 6: develop an evaluation plan

**Figure 3:**
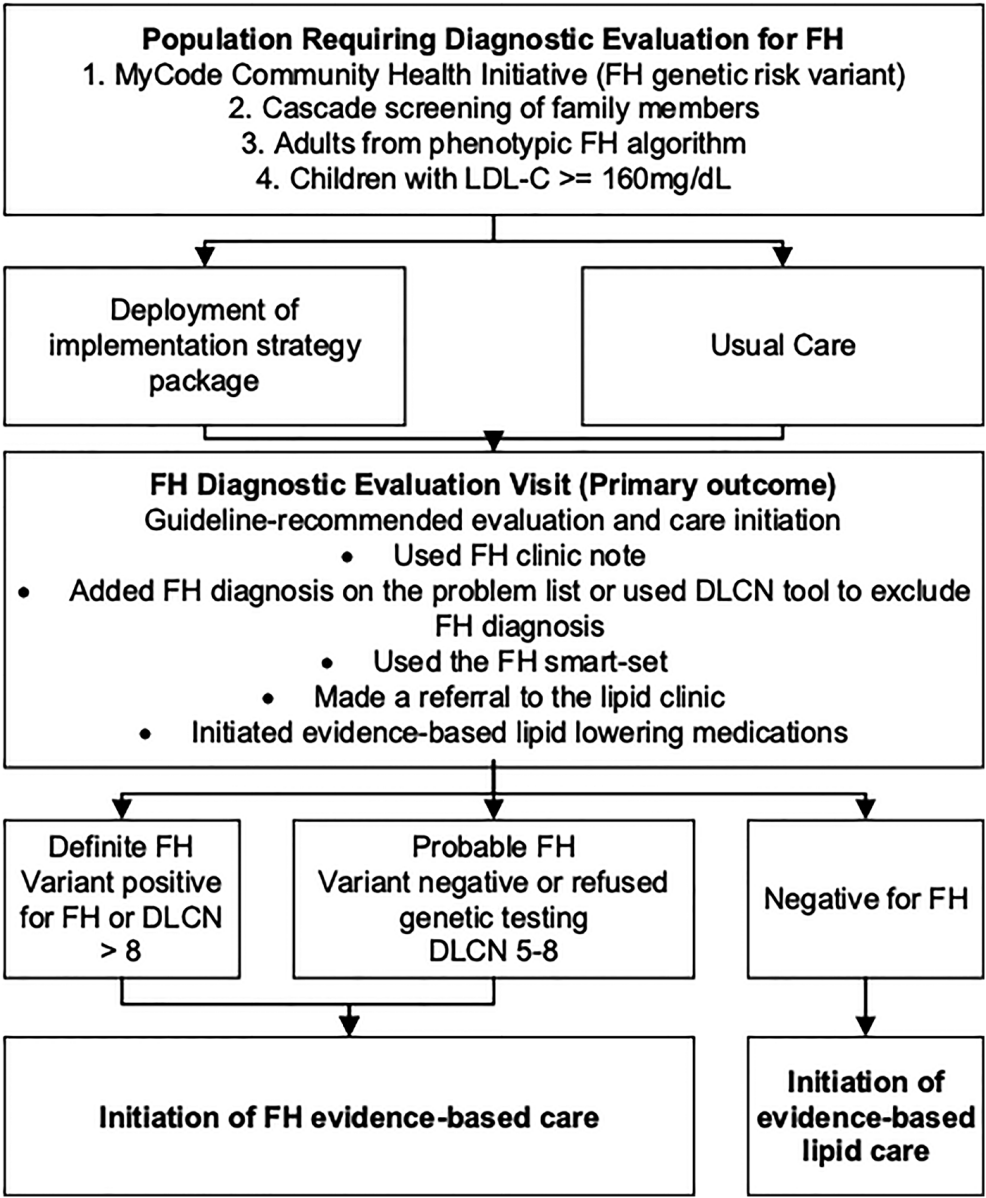
CARE-FH study diagnostic evaluation plan. Reproduced under the CC-BY license from Jones LK et al. (49).

An evaluation plan was developed using the Conceptual Model for Implementation Research and included the following implementation outcomes: adoption, penetration, acceptability, feasibility, fidelity, sustainability, and cost (51, 52). The study team has adapted this model to CARE-FH (Figure 3). The evaluation plan also includes service and health outcomes are detailed in Table 9.

**Table 9:** CARE-FH study evaluation plan. Reproduced under the CC-BY license from Jones LK et al. (51).

Domain	Aim	Outcome	Construct measured	Data source
Implementation	2	**Adoption**	**FH diagnostic evaluation defined as completed of one of the following:** - Used FH clinic note to document care - Added FH diagnosis on the problem list or used DLCN tool to exclude FH diagnosis - Used the FH smart-set (i.e., ordered a genetic test for FH) - Made a referral to the lipid clinic - Initiate evidence-based lipid lowering medications	EHR, administrative data
		Penetration	Proportion of the primary care clinicians that completed the five components of the FH diagnostic evaluation compared to those that did not use it.	
	3	Acceptability	Clinician and patient satisfaction and self-efficacy with the implementation strategy package	Semi-structured interviews
		Cost	Cost to implement the implementation strategy package	Micro-costing
		Feasibility	Clinician adoption and penetration for completion of the FH diagnostic evaluation and measured utility of implementation strategy package	Semi-structured interviews and EHR data
		Fidelity	Documentation of adaptations to the FH diagnostic evaluation program	Checklist, direct observation
		Sustainability	Potential for institutionalization	Surveys, Advisory board consultation
Service	4	Timeliness	Time to: FH screen, completion of diagnostic evaluation, medication initiation	EHR, administrative data
Health		Safety	Medication-related side effects	
		Intermediate	LDL-C reduction	
		Process	Return of genetic result	
			Initiation of cascade testing	

EHR, electronic health record; FH, familial hypercholesterolemia; LDL-C, low-density lipoprotein cholesterol.

Bolded is the primary outcome of the study.

## 4. Discussion

The application of principles from implementation science to the field of FH has been discussed in many recent articles including original research, reviews, commentaries, and guidelines (53–63). By leveraging implementation science, which aims to close the large gap from knowledge generation to implementation into practice, we can improve every component of FH care.

Intervention mapping provides a systematic process for developing evidence-based implementation strategies to improve FH care. Through steps 1–3 in this process, we identified barriers and facilitators to three components of FH care: identification, cascade testing, and management. We also found that barriers and facilitators may not be one-dimensional and exist at the patient, clinician, and health system levels. To overcome these barriers, we need to develop an implementation strategy package that addresses each level and component of FH care. We have provided a list of implementation strategies specific to FH care that others can adapt to their local context. For steps 4–6, we highlighted an example of a currently funded study, CARE-FH, that is using implementation strategies based on evidence to improve FH identification. This systematic evaluation using intervention mapping allowed us to develop implementation strategies and allows other teams to replicate and/or adapt these strategies by other teams. The FH specific strategies that were developed in this study can be tailored by others to their specific context to improve care.

To date, there have been many explorations of barriers and facilitators to FH care, but few have developed strategies to address them that can then be implemented into practice and subsequently evaluated (10). Previous studies focused on only one component of FH care or at one level (patient, clinician, or health system) (53, 64). By addressing these barriers for components of FH care at multiple levels, we can more thoroughly address the problems faced by individuals with FH and their families.

Due to the limited evidence on implementation strategies to improve FH care, two recent review articles have retrospectively mapped interventions from previous studies to a compilation of implementation strategies (53, 65). This work has facilitated the use of a common language for naming and describing implementation strategies. By having a common nomenclature, it becomes easier to tailor implementation strategies for specific contexts such as FH care. From these two articles, we know that only certain implementation strategies, including assess for readiness and identify barriers and facilitators, develop and organize quality monitoring systems, create new clinicals teams, facilitate relay of clinical data to providers, and involve patients and family members, have been tested in practice for FH care (53). There is a need to deploy other implementation strategies listed in compilations such as promote network weaving, create a learning collaborative, change liability law, among others (53, 65). In addition, implementation strategies need to be explained using a standardized reporting method so they can be replicated in the future (53, 65, 66).

Some cholesterol and FH guidelines have started to include sections on how to help improve the translation of their guidelines into practice (1, 60–63, 67). However, these guidelines are not formatted in such a way as to promote their translation and implementation in the clinic setting (54). A recent editorial provides a framework to help facilitate the translation of evidence-based recommendations with implementation recommendation to create clinical practice guidelines that can then be implemented and evaluated in local contexts (55).

### 4.1. Limitations

An important limitation is that not all health systems, clinicians, or patients will have the ability to implement strategies that affect multiple levels or multiple components of FH care. It will be important to identify strategies that are relevant to specific health contexts and the needs of particular health systems. This project only reported implementation strategies that we have found important for our work in our health care context, but other strategies might arise or need to be adapted. Another limitation is that this study only reported on the ERIC compilation of strategies that were relevant for the implementation phase of a study and not those that are important for pre-implementation work. Steps 1 and 2 of intervention mapping include strategies relevant for pre-implementation, including conducting a needs assessment, identifying barriers and facilitators, and assessing readiness of the organization to implement the evidence-based practice. Barriers and facilitator data collected from FH patients was supplemented with the literature to account for broader perspectives. Additional pre-implementation strategies that should be considered prior to implementation include developing evaluative and iterative strategies (e.g., developing and organizing quality monitoring systems) and adapting and tailoring strategies to the local context.

## 5. Conclusions

Using a systematic, evidence-based, multilevel approach to the development of implementation strategies, implementation recommendations, and evaluation is imperative to success in changing practice and care for individuals with FH. This study provides an overview of one evidence-based approach to accomplish this task: intervention mapping. The implementation strategies developed as part of this report can be utilized by others to improve FH care and learnings from the highlighted study can facilitate near-term deployment into practice as well as evaluation of both clinical and implementation outcomes.

## Data Availability

The original contributions presented in the study are included in the article/**Supplementary Material**, further inquiries can be directed to the corresponding author.
