# Supplementary material for: Designing implementation strategies to improve identification, cascade testing, and management of families with familial hypercholesterolemia: An intervention mapping approach
Source: Front Health Serv. 2023 Apr 28;3:1104311. doi: 10.3389/frhs.2023.1104311 (PMC10175779; doi:10.3389/frhs.2023.1104311)
Supplement: Supplementary file 1 [file Datasheet1.pdf]

Supplementary Material

1 Supplementary Files

Supplementary File 1. Dyadic Interview Guide

Supplementary File 2. Geisinger Survey for Individuals with FH

Supplementary File 3. Family Heart Foundation Survey for Individuals with FH

Supplementary File 4. Family Member Survey

Supplementary File 1. Dyadic Interview Guide

Introduction: Thank you for agreeing to help with this quality improvement process. As a reminder, your participation in helping us improve the quality of our care and resources is voluntary. As you recall, we expect this interview to take 45 minutes to an hour. Your responses will help us improve how we communicate with individuals and families about risks related to FH. I will ask you some broad questions and then follow up with some more specific questions about the materials you were sent for homework (the "Dear Family" letter, chatbots, and description of the Direct Contact Program). Have you each reviewed the materials that were sent before to your emails?

If yes: proceed to next section.

If no: need to reschedule until they have reviewed the materials.

Everything you tell me will be kept confidential. This means that your interview responses will only be shared with research team members. When we write our report on this and the other interviews we are conducting, nothing in our report will identify you. Please be honest with your responses. You can say whatever you want – nothing will hurt my feelings and nothing you say will have a negative effect on your care. We will record this conversation, but the transcript from the conversation will not include any information that identifies you. Please remember: you don't have to talk about anything you don't want to talk about. You can decline to answer any question and you may end the interview at any time.

Is it OK if I start recording now?

**Stem**	**Probe**	**Concept/Modality**
- How did your family talk about your FH diagnosis?	- (to proband) How did you share your FH results with (family member name) in the interview today?<br>- (to family member) How did you respond when (proband name) told you about this?	Warm up – Disclosure of Result
- How does your family talk about their FH diagnosis? (e.g., risks for heart disease, having or managing high cholesterol, passing the gene down, etc.)	- How does your family feel about your/their FH diagnosis?<br>- What's made it easier to share this information with family members?<br>- What's made it difficult to share this information with family members?	Family Health Communication of FH Norms
- If you were looking for resources or information on FH to help you talk to family members about FH, where would you look for these resources?	- What information would you want to share with your relatives who may have inherited FH?	Information Needs
[Description of Direct Contact - Stress that proband has to give consent, relative's name and contact info, and provider would only contact those the proband wanted]<br>- What do you think about having a provider contact relatives directly to talk about your FH result?	- What type of provider would you want to do this? (e.g., nurse, genetic counselor, PCP/GP, navigator trained for this purpose etc.)<br>o Can you explain what makes you choose that provider?<br>o How would you feel about a trained representative or healthcare provider from an organization like the FH	Direct Contact

	Foundation contacting relatives about your FH result? (may need to explain FH Foundation)<br>- Think about how you and your family members communicate about FH. What would be the best thing for your [preferred provider] to say when they contact your family members to discuss FH?<br>o Is there information a provider should NOT share that should only come from a relative?<br>o How would you (FH proband) want to be involved with a provider sharing this information with family members? (e.g., go over script of what to tell relative with provider, discuss when to contact family members, etc.)<br>- What should a provider say to at-risk family members to prompt action?<br>- What should a provider NOT say to at-risk family member to prompt action?	
- How would family members respond if they were contacted by a provider?	- Which of your family members would respond best to this method of contact? (e.g., specific people in family and/or certain family relationships like siblings, children, etc.)<br>- Why might this work best for those family members?<br>- Who would this NOT work for in your family?<br>o Can you tell me more about why this might not work [person/people]?	Direct Contact
- Ideally, if a provider were to help share this information with family – what should the process be? what would you want this to look like?	- How would you feel if a provider sent a letter first and then followed up with a phone call for the Direct Contact Program? (letter would be different from "Dear Family" letter)<br>- What approach might work better for you/your family?<br>- What contact information for relatives would you be willing to provide? (e.g., phone number, email address, mailing address, etc.)	Direct Contact

	- What would make it easier for family members to do follow up testing?<br>- How else would you want a provider to help?	
[Describe CASCADE program]<br>- Geisinger has a program called CASCADE that you read a little material on. This program include genetic counseling and allows family members to order an FH genetic test via a mail order kit to do at home and send back. Family members could choose this option in talking to a provider during Direct Contact. If this was offered to you (family members) how would you respond?	- How would other family members respond to this option?<br>- How can we improve how we talk about this program with at-risk family members?<br>o How would you feel if you had the option to order a genetic test (mail order kit)?<br>o How would you want to order a genetic test (mail order kit)? (e.g., have provider order for you, order online, etc.)<br>- What other information on this option would you want as a proband/relative?	Direct Contact
Transition to Letter	Thank you for sharing your thoughts on a Direct Contact program. We'd now like to switch topics slightly and get your thoughts on the "Dear Family" Letter. This was sent to you via email. It may help if you are able to look at it or your notes on it while we go through questions.	
- (To proband) How would you feel using this letter to help you share your results?	- Please tell me more about why/why not.<br>- How would you use it? (e.g., mail it, use it to guide conversation, etc.)	"Dear Family" Letter
- (To family member) How would you feel receiving this letter?	- What would you do next if you got this letter? (e.g., talk to doctor, talk to family, etc.)<br>- What can we change about the letter to help you talk about your relative's result with your doctor?<br>o What could we improve about the letter to make you and other family members follow up with testing?<br>- What questions would you ask your relative if you received this letter?	"Dear Family" Letter
- (To Both) How could we make the letter better?	- What can we improve to make it more likely someone will take action to follow up and pursue testing?<br>- What other information would you want from the letter?<br>- Can you explain what type of information that is? Why?	"Dear Family" Letter

Transition to Chatbot	Thank you for sharing your thoughts on the “Dear Family” Letter. We’d now like to move to our last topics and get your thoughts on the chatbots. The links for the chatbots were sent to you via email. It may help if you are able to look them or your notes on them while we go through questions.	
- (To proband) How would you feel about using a chatbot to share your results?	- Please tell me more about why/why not.<br>- If you were to use a Chatbot to share your results with family, how would you do that? (i.e., how to send, timing of sending, contacting relative before sending, etc.)	Chatbot
- (To family member) How would you feel receiving the chatbot?	- What questions would you ask of your relative if you received this?<br>- What would you do next if you got the chatbot? (e.g., talk to doctor, talk to family, look up info online, etc.)<br>- How comfortable would you be if after the chatbot gave information on FH, it gave you the option to order a genetic test by mail?<br>- How can we improve/enhance the chatbot to help you talk about your relative’s result with your doctor?	Chatbot
- (To both) How could we make this tool better for both of you?	- How would you feel if you received reminders from the chatbot on this information?<br>o How often would you want reminders?<br>o How many reminders would be too many?<br>o When should these reminders come in?<br>- What other information would you want from the chatbot?<br>o Is there information that should NOT come from the chatbot and only from a healthcare provider? Relative?<br>▪ Can you explain what type of information that is? Why?	Chatbot
- How would you feel having a combination of resources, like the letter, chatbot, and provider help to share this information?	- What combination do you think is best?<br>- Would you want to use different communication resources at different	Combination of Modalities

	times as you share this information with family?<br>- Would you use different communication resources with different family members? If so, How?<br>- As a family member, how would you respond to receiving a combination of these resources?<br>o As a family member, what would work best for you?<br>- Please tell me more about why X combination would work best.	

Supplementary File 2. Geisinger Survey for Individuals with FH

IMPACT-FH: Geisinger Survey for Individuals with FH

Welcome and Instructions

Greetings!

Geisinger and the FH Foundation, a non-profit research and advocacy organization, want to learn how to better support people as they share information about Familial Hypercholesterolemia (FH) with their relatives.

Your responses will help us improve our current resources and design new ways for people to talk about FH with their family.

During the survey, you will be asked to:

· Review a letter
· Watch a video of a chatbot
· Read about a new program to help families share FH information
· Answer written response and multiple-choice questions about these resources

It should take you about 25 minutes to review the resources and complete the survey. We ask that you be as detailed as possible when answering the written response questions.

Complete the survey by Monday, September 28, 2020, to be entered into a gift card drawing. Five (5) individuals who complete the survey will be chosen at random and will each receive a $50 Amazon gift card. **You must enter your contact information when asked in the survey. We will notify you via email if you win a gift card. Odds of winning a gift card are based on the total number of individuals who complete the survey.**

At the end of the survey, you will be invited to share a similar survey with your blood relatives and your spouse or partner. Please note that the gift card drawing does not apply to your family members/partner/spouse who complete that survey.

Choosing not to take the survey will have no effect on your current medical care. If you choose to take the survey, you may exit it at any time.

Click the "Next" button at the bottom of the screen to start the survey.

If you have questions, concerns, or problems with the survey, please call our team at 1-866-910-6486, Option 2. You may also email us at IMPACTFH@geisinger.edu.

Thank you, we sincerely value your input.

Geisinger | FH FOUNDATION

IMPACT-FH: Geisinger Survey for Individuals with FH

Demographic Information

Please answer the following questions.

* 1. Please type your age:

* 2. What is your biological sex?

- Male
- Female
- Prefer not to say

* 3. What is your annual household income?

- < $25,000
- $25 – 50,000
- $50 – 75,000
- $75 – 100,000
- > $100,000
- Prefer not to answer

* 4. What is your highest level of school completed?

- Some High School
- Graduated High School/GED
- Some College
- Associate’s Degree
- Bachelor’s Degree
- Graduate/Professional Degree
- Prefer not to answer

* 5. What state do you currently live in?

State/Province -- select state --

* 6. How did you find out that you have FH? (check all that apply)

- [ ] Result from MyCode
- [ ] Other genetic testing
- [ ] Diagnosed by doctor
- [ ] Other, please explain:

* 7. Were you the first person in your family to be diagnosed with FH?

- ( ) Yes
- ( ) No

IMPACT-FH: Geisinger Survey for Individuals with FH

Demographic Information - FH Diagnosis Age

Please answer the following question.

* 8. Please type in the age when you were first diagnosed with FH:

IMPACT-FH: Geisinger Survey for Individuals with FH

Demographic Information - FH Diagnosis Year

Please answer the following question.

* 9. When was the first person in your family diagnosed with FH? Please type year or your best estimation:

IMPACT-FH: Geisinger Survey for Individuals with FH

FH Infographic

Please review the infographic below, then click "Next" at the bottom of the page to continue the survey.

Familial Hypercholesterolemia (FH)

FH is Common

FH is a **genetic disorder** that causes dangerously **high levels of LDL ("bad") cholesterol** from birth, leading to early heart disease.

FH affects **1 in 250 people or 30 million worldwide** of all races and ethnicites.

FH is highly underdiagnosed - **90% of people with FH don't know they have it.**

FH Causes Early Heart Disease

~17,500 – the same number of people die from FH every year as from car accidents.

~790,000 Americans a year have a heart attack. Untreated individuals with FH have a **20X increased risk** of a heart attack.

FH is Important to Find

Consider screening for FH if you have a family history of high cholesterol and/or early heart disease.

FH can be diagnosed clinically or with a genetic test.

Genetic testing for FH should include **pre- and post-genetic counseling**.

Learn more and get support at
www.theFHfoundation.org

FH Family

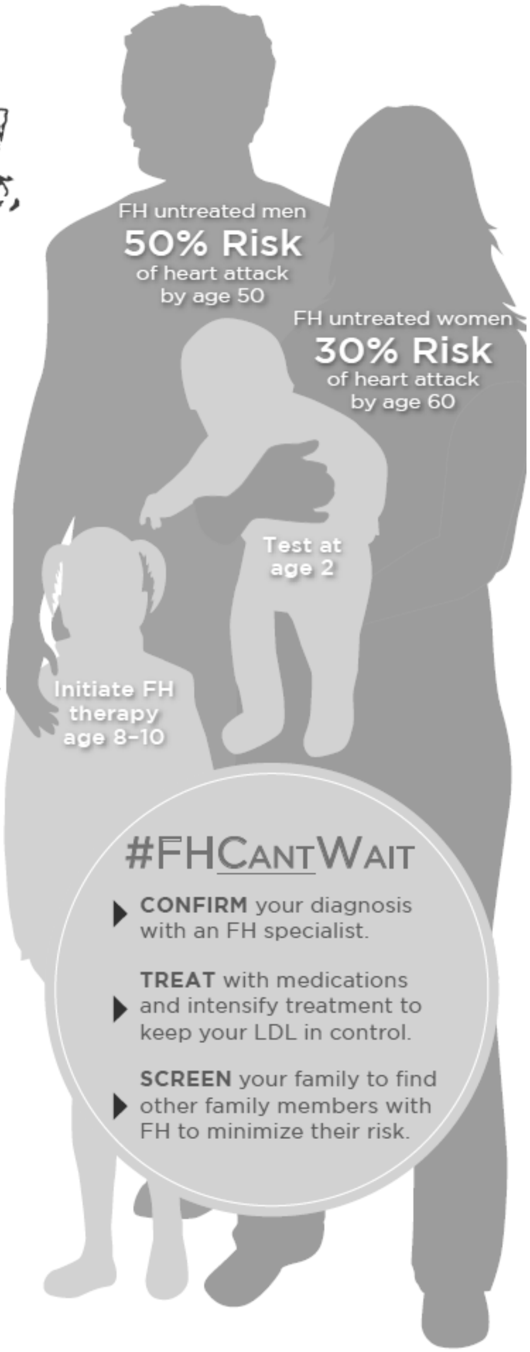

#FHCantWait

- **CONFIRM** your diagnosis with an FH specialist.
- **TREAT** with medications and intensify treatment to keep your LDL in control.
- **SCREEN** your family to find other family members with FH to minimize their risk.

FH Foundation
a 501(c)(3) non-profit research and advocacy organization

© 2018, The FH Foundation. All rights reserved. 07/18

Raising Awareness. Saving Lives.

IMPACT-FH: Geisinger Survey for Individuals with FH

Section 1: Dear Family Letter

The Dear Family Letter is a resource that was developed at Geisinger. It is given to people who receive a genetic diagnosis of FH through Geisinger's MyCode Community Health Initiative to help share their result with relatives of their choosing.

Please read the sample Dear Family Letter below before moving on to the next page.

You may return to this page at any time to review the Dear Family Letter by using the "Back" button at the bottom of the page. Your responses will be saved if you go back to review the letter.

6/17/2020

Dear Ms. Jane Doe,

I learned that my *LDLR* gene does not work correctly through the Geisinger MyCode study. This gene change puts me at higher risk for early heart attack and stroke from inherited high cholesterol. Another name for this genetic risk is Familial Hypercholesterolemia (FH).

Gene changes run in families. Parents, children, brothers and sisters of people with a *LDLR* gene change have a 50% chance of having the same gene change and health risks. Other family members (aunts, uncles, nieces, nephews, cousins, grandchildren) may also have the same gene change and health risks.

People who have FH often need extra medical care. This extra care may include looking for and treating potentially life-threatening heart problems.

A simple "yes/no" blood or saliva test can tell you if you also have the same gene change and health risks.

You may be able to get this "yes/no" test for free or at lower-cost from the same lab that ran my test. Free genetic testing for family members is offered for 90 days after **6/10/2020**, my report date. The appointment with the provider to order this test will be billed to you or your insurance.

<u>**What you should do next**</u>:

- **Call the Geisinger MyCode Genomic Screening and Counseling team (toll-free) at 1-844-250-8031.** They can schedule an appointment to start the "yes/no" testing process. They can also answer your questions.

- **If you do not live in PA, you can find a genetic counselor to order this test and discuss your risks at:** https://www.nsgc.org/page/find-a-genetic-counselor

The healthcare provider ordering your test will need this information:

c.2054C>T p.Pro685Leu in the *LDLR* gene (NM_000527.5)
Laboratory: Invitae.

This letter gives my permission for Geisinger to share my genetic test results with you for your care. <u>**Please bring this letter with you to your appointment.**</u>

If you have any questions or concerns, please call the Geisinger MyCode team (toll-free) at **1-844-250-8031**.

Sincerely,

____

Mr. John Doe

INVITAE

SECONDARY FINDINGS SCREEN RESULTS

**Patient name:**	John Doe	**Sample type:**	gDNA	**Report date:**	
**DOB:**		**Sample collection date:**		**Invitae #:**	
**Sex:**	Male	**Sample accession date:**		**Clinical team:**	
**MRN:**					

Test performed

Sequence analysis and deletion/duplication testing of the 59 genes listed in the Genes Analyzed section.

- Secondary Findings Add-on

RESULT: POSITIVE

A clinically significant genetic change was found in the LDLR gene, which is associated with a heart-related condition.

GENE	VARIANT	ZYGOSITY	VARIANT CLASSIFICATION
LDLR	c.2054C>T (p.Pro685Leu)	heterozygous	PATHOGENIC

About this test

This test evaluates 59 genes for variants (genetic changes) that indicate a significantly increased risk of developing certain types of cancer, heart-related conditions, or other types of actionable medical genetic conditions. These are disorders for which effective medical interventions and preventive measures are known and available. Genetic changes of uncertain significance are not included in this report; however, if additional evidence becomes available to indicate that a previously uncertain genetic change is clinically significant, Invitae will update this report and provide notification.

Next steps

- This is a medically important result that should be discussed with an appropriate healthcare provider. Genetic counseling is recommended to discuss the implications of this result and potential next steps.
- Consider sharing this result with relatives as they may also be at risk. Details on our Family Variant Testing program can be found at www.invitae.com/family.
- Register your test at www.invitae.com/patients to download a digital copy of your results. You can also access educational resources about how your results can help inform your health.

Clinical Summary

A Pathogenic variant, c.2054C>T (p.Pro685Leu), was identified in LDLR.

IMPACT-FH: Geisinger Survey for Individuals with FH

Section 1: Dear Family Letter

Please answer the following questions.

You may use the "Back" button at the bottom of the page to return to the sample Dear Family Letter. Your responses will be saved if you go back to review the letter.

* 10. Do you consider this letter to be a reliable/trustworthy source for medical information?

- ◯ Yes
- ◯ No
- ◯ I'm not sure
- ◯ Other, please explain:

* 11. How easy is it to understand the information in the letter?

- ◯ Very easy
- ◯ Somewhat easy
- ◯ Neutral
- ◯ Somewhat difficult
- ◯ Very difficult

* 12. How should we make this letter available to you to share with your relatives? *(Check all that apply)*

- ☐ Share it to my email
- ☐ Share it to my patient portal (e.g., MyGeisinger, MyChart, etc.)
- ☐ Mail paper copies to me
- ☐ Other, please explain:

* 13. Would you send this type of letter to a relative?

- Yes
- No

IMPACT-FH: Geisinger Survey for Individuals with FH

Section 1: Dear Family Letter

Please be detailed in your responses to the below question, so we can better understand how to improve our resources for individuals and families with FH.

You may use the "Back" button at the bottom of the page to return to the sample Dear Family Letter. Your responses will be saved if you go back to review the letter.

* 14. Why would you NOT send this letter to relatives?

IMPACT-FH: Geisinger Survey for Individuals with FH

Section 1: Dear Family Letter

Please answer the following questions.

You may use the "Back" button at the bottom of the page to return to the sample Dear Family Letter. Your responses will be saved if you go back to review the letter.

* 15. Who would you send this type of letter to? *(Check all that apply)*

- [ ] Mother
- [ ] Father
- [ ] Sister(s)
- [ ] Brother(s)
- [ ] Son(s)
- [ ] Daughter(s)
- [ ] Grandchild(ren)
- [ ] Grandparents – mother’s side
- [ ] Grandparents – father’s side
- [ ] Cousin(s) – mother’s side
- [ ] Cousin(s) – father’s side
- [ ] Aunt(s) – mother's side
- [ ] Aunt(s) – father's side
- [ ] Uncle(s) – mother’s side
- [ ] Uncle(s) – father’s side
- [ ] Other, please specify:

* 16. Why would you send the letter to these people? *Please be detailed in your response.*

IMPACT-FH: Geisinger Survey for Individuals with FH

Section 1: Dear Family Letter

Please be detailed in your responses to the below questions, so we can better understand how to improve our resources for individuals and families with FH.

You may use the "Back" button at the bottom of the page to return to the sample Dear Family Letter. Your responses will be saved if you go back to review the letter.

* 17. What other information do you think could be included in the letter to help your relatives take next steps to find out if they have FH?

18. What else do you want to share with us about the letter and/or how to make it better?

Geisinger | FH FOUNDATION

IMPACT-FH: Geisinger Survey for Individuals with FH

Section 2: Chatbot

A chatbot is an online conversational tool. People with FH are offered a chatbot to help them more easily share information about FH with relatives. Relatives of the individual’s choosing are sent the chatbot to help them learn more about FH and their risks.

Please watch the chatbot video below before moving on to the next page.

If you are having trouble playing this video within the survey, please copy and paste the following link *in a new tab or window*: https://youtu.be/aTDKdodrIzs

You may return to this page at any time to re-watch the chatbot video by using the "Back" button at the bottom of the page. Your responses will be saved if you go back to re-watch the video.

IMPACT-FH: Geisinger Survey for Individuals with FH

Section 2: Chatbot

Please answer the following question.

You may use the "Back" button at the bottom of the page to return to the chatbot video. Your responses will be saved if you go back to re-watch the video.

* 19. Would you send this type of chatbot link to any of your relatives?

○ Yes

○ No

IMPACT-FH: Geisinger Survey for Individuals with FH

Section 2: Chatbot

Please answer the following question.

You may use the "Back" button at the bottom of the page to return to the chatbot video. Your responses will be saved if you go back to re-watch the video.

* 20. Why would you NOT send the chatbot to any of your relatives? *(Check all that apply)*

- [ ] I am not comfortable with technology
- [ ] My relatives are not comfortable with technology
- [ ] I would prefer to call or tell them myself
- [ ] Other, please explain:

IMPACT-FH: Geisinger Survey for Individuals with FH

Section 2: Chatbot

Please answer the following questions.

You may use the "Back" button at the bottom of the page to return to the chatbot video. Your responses will be saved if you go back to re-watch the video.

* 21. Who would you send the chatbot link to? *(Check all that apply)*

- ☐ Mother
- ☐ Father
- ☐ Sister(s)
- ☐ Brother(s)
- ☐ Son(s)
- ☐ Daughter(s)
- ☐ Grandchild(ren)
- ☐ Grandparents – mother's side
- ☐ Grandparents – father's side
- ☐ Cousin(s) – mother's side
- ☐ Cousin(s) – father's side
- ☐ Aunt(s) – mother's side
- ☐ Aunt(s) – father's side
- ☐ Uncle(s) – mother's side
- ☐ Uncle(s) – father's side
- ☐ Other, please specify:

* 22. Why would you send the chatbot to these people? *Please be detailed in your response.*

IMPACT-FH: Geisinger Survey for Individuals with FH

Section 2: Chatbot

Please answer the following question.

You may use the "Back" button at the bottom of the page to return to the chatbot video. Your responses will be saved if you go back to re-watch the video.

* 23. When would you send the chatbot link to relatives?

- ◯ Call/talk first, then send the link
- ◯ Send the link immediately
- ◯ Some I'd call/talk to first, some I'd just send the link
- ◯ Other

IMPACT-FH: Geisinger Survey for Individuals with FH

Section 2: Chatbot

Please answer the following question.

You may use the "Back" button at the bottom of the page to return to the chatbot video. Your responses will be saved if you go back to re-watch the video.

* 24. Please explain when you would send the chatbot link to relatives. *Please be detailed in your response.*

Section 2: Chatbot

Please answer the following questions.

You may use the "Back" button at the bottom of the page to return to the chatbot video. Your responses will be saved if you go back to re-watch the video.

* 25. Would you wait to send the chatbot until after you spoke with a doctor or a genetic counselor?

- ◯ Yes
- ◯ No
- ◯ I'm not sure
- ◯ Other, please explain:

* 26. Select the options you feel comfortable using to share the chatbot. *(Check all that apply)*

- ☐ Text message
- ☐ Email
- ☐ Facebook messenger
- ☐ Other, please specify:

* 27. What would influence how you share the chatbot with each relative? *(Check all that apply)*

- [ ] How well I know the relative
- [ ] How closely related we are
- [ ] Contact information I have for relatives
- [ ] How I think my relative would like to receive this information
- [ ] Things I know about my relative's mental health
- [ ] Things I know about my relative's physical health
- [ ] My relative's attitude toward health/healthcare
- [ ] My relative's comfort level with technology
- [ ] Other, please explain:

* 28. Do you consider the chatbot to be a reliable/trustworthy source for medical information?

- ( ) Yes
- ( ) No
- ( ) I'm not sure
- ( ) Other, please specify:

* 29. How easy is it to understand the information in the chatbot?

- ( ) Very easy
- ( ) Somewhat easy
- ( ) Neutral
- ( ) Somewhat difficult
- ( ) Very difficult

IMPACT-FH: Geisinger Survey for Individuals with FH

Section 2: Chatbot

Please be detailed in your responses to the below questions, so we can better understand how to improve our resources for individuals and families with FH.

You may use the "Back" button at the bottom of the page to return to the chatbot video. Your responses will be saved if you go back to re-watch the video.

* 30. What can we improve about the chatbot to make it more likely you would share the chatbot with your relatives?

* 31. What can we improve about the chatbot to make it more likely your relatives will take action?

32. What else do you want to share with us about the chatbot and/or how to make it better?

IMPACT-FH: Geisinger Survey for Individuals with FH

Section 3: Direct Contact Program

The Direct Contact Program is a new program being designed by Geisinger and the FH Foundation. It will be another way for individuals diagnosed with FH to share their diagnosis with the relatives of their choosing. The Direct Contact Program has not been finalized yet. The description below is a general idea of what the program will look like.

Please read the information below before moving on to the next page.

You may return to this page at any time to review this information by using the "Back" button at the bottom of the page. Your responses will be saved if you go back to review this information.

What is a Direct Contact Program?

Step 1

A person gets tested, via genetic testing and/or cholesterol testing, and finds out they have Familial Hypercholesterolemia (FH).

Step 2

The person with FH gives a healthcare provider permission to share their FH diagnosis with their at-risk relatives.

- The person with FH can pick which at-risk relatives they want the healthcare provider to contact and gives the healthcare provider contact information (for example, address, email, telephone number) for each of those relatives.

Step 3

The healthcare provider contacts at-risk relatives to share that their family member has FH and to explain their risks of heart disease if they have FH, too.

Step 4

A healthcare provider, like a genetic counselor or doctor, can counsel relatives and help them get testing.

Why would someone use a Direct Contact Program?

Sharing complex health information about FH with at-risk relatives can be hard.

Talking to a healthcare provider can motivate at-risk relatives to get tested for FH.

If relatives test positive for FH, they can get care for their FH health risk sooner.

IMPACT-FH: Geisinger Survey for Individuals with FH

Section 3: Direct Contact Program

Please answer the following questions.

You may use the "Back" button at the bottom of the page to return to the information about the Direct Contact Program. Your responses will be saved if you go back to review the information.

* 33. Imagine if Geisinger offered a Direct Contact Program. In this program, you would provide the name and contact information of some, or all, of your relatives at risk for FH. With your permission, a healthcare provider from the program would contact your relatives to tell them that "A family member has received an FH diagnosis and you could be at risk too."

Using a 1-5 scale, where 1 means Not at All Likely and 5 means Extremely Likely, indicate how likely you would be to use such a Direct Contact Program.

1. Not at All Likely	2. Somewhat Unlikely	3. Neutral	4. Somewhat Likely	5. Extremely Likely
◯	◯	◯	◯	◯

* 34. If you used this Direct Contact Program to share information about FH with your at-risk relatives, would you want the provider to share your name and your FH result?

- ◯ Yes
- ◯ No
- ◯ Unsure
- ◯ Depends on the relative

* 35. How helpful would it be if a healthcare provider offered to contact your relatives on your behalf to inform them of your FH diagnosis and their potential risk and need for screening?

- ◯ Very helpful
- ◯ Somewhat helpful
- ◯ Neutral
- ◯ Somewhat unhelpful
- ◯ Not helpful at all
- ◯ Unsure
- ◯ Other (please specify)

* 36. What type of healthcare provider would you feel comfortable directly contacting your relatives? *(Check all that apply)*

- [ ] Genetic counselor (a healthcare provider with expertise in genetics)
- [ ] Nurse
- [ ] Primary care provider
- [ ] Specialist (e.g., cardiologist, lipidologists, etc.)
- [ ] Pharmacist
- [ ] Medical staff trained for this purpose
- [ ] Other, please specify:

* 37. Who would you want the Direct Contact Program to contact on your behalf?

- ◯ All my at-risk relatives
- ◯ Only my at-risk relatives of my choosing
- ◯ None of my at-risk relatives

IMPACT-FH: Geisinger Survey for Individuals with FH

Section 3: Direct Contact Program

Please answer the following question.

You may use the "Back" button at the bottom of the page to return to the information about the Direct Contact Program. Your responses will be saved if you go back to review the information.

38. How would you decide which relatives to use Direct Contact for? *(Check all that apply)*

- [ ] How well I know the relative
- [ ] Contact information I have for relative
- [ ] How I think my relative would like to receive this information
- [ ] Things I know about my relative’s mental health
- [ ] Things I know about my relative’s physical health
- [ ] How closely related we are
- [ ] My relative’s attitude toward health/healthcare
- [ ] Other, please explain:

IMPACT-FH: Geisinger Survey for Individuals with FH

Section 3: Direct Contact Program

Please answer the following questions.

You may use the "Back" button at the bottom of the page to return to the information about the Direct Contact Program. Your responses will be saved if you go back to review the information.

* 39. Think about the at-risk relatives you would want the Direct Contact Program to contact. What contact information for your relatives would you be comfortable providing? *(Check all that apply)*

- [ ] Phone number
- [ ] Mailing address
- [ ] Email address
- [ ] Other, please explain:

* 40. How acceptable do you think it would be to your at-risk relatives to receive information about your FH diagnosis directly from an FH expert in the Direct Contact Program?

- ○ It would be very acceptable to most of my relatives
- ○ Most would accept it, but a few would not accept it
- ○ In general, my relatives would find it neither acceptable nor unacceptable
- ○ A few would accept it, but most would not accept it
- ○ None of my relatives would accept it
- ○ Unsure

Geisinger | FH Foundation

IMPACT-FH: Geisinger Survey for Individuals with FH

Section 3: Direct Contact Program

Please be detailed in your responses to the below questions, so we can better understand how to improve our resources for individuals and families with FH.

You may use the "Back" button at the bottom of the page to return to the information about the Direct Contact Program. Your responses will be saved if you go back to review the information.

* 41. Ideally, how would you want a healthcare provider to help you share information with relatives about their FH risks?

42. What else do you want to share with us about the Direct Contact Program and/or how to make it better?

* 43. If you did not want to use the letter, chatbot, or the Direct Contact Program to share your FH result with your at-risk relatives, how would you inform these family members?

Geisinger | FH FOUNDATION

IMPACT-FH: Geisinger Survey for Individuals with FH

Gift Card Drawing and Additional Opportunities

* 44. **Five (5) individuals** who complete the survey by **Monday, September 28, 2020,** will be chosen at random and will each receive a **$50 Amazon gift card**. You must enter your contact information on the next page in order to be in the drawing. We will notify you via email if you win a gift card. Odds of winning a gift card are based on the total number of individuals who complete the survey.

Would you like to be entered into the gift card drawing?

◯ Yes

◯ No

* 45. Would you be interested in taking part in further opportunities on how healthcare providers can support families talking about FH?

◯ Yes

◯ No

IMPACT-FH: Geisinger Survey for Individuals with FH

Gift Card Drawing and Additional Opportunities

* 46. Please provide your name, email address, and phone number below:

Name

Email Address

Phone Number

<u>Share a Survey with your Family Members!</u>

We are also interested in understanding how *<u>your family members</u>* feel about these materials.

If you are willing to share a similar survey with your blood relatives, or your spouse/partner, *<u>please copy the link below and share it with them</u>*.

Please note that the gift card drawing does not apply to your family members/partner/spouse who complete that survey.

You may share the link with your family members in any way you choose.

<u>Family Member Survey Link:</u>

https://www.surveymonkey.com/r/IMPACTFH_Family2

Supplementary File 3. Family Heart Foundation Survey for Individuals with FH

IMPACT-FH: FH Foundation Survey for Individuals with FH

Welcome and Instructions

Greetings!

The FH Foundation and Geisinger, a health system in Pennsylvania, want to learn how to better support people as they share information about Familial Hypercholesterolemia (FH) with their relatives.

Your responses will help us improve our current resources and design new ways for people to talk about FH with their family.

During the survey, you will be asked to:

· Review a letter
· Watch a video of a chatbot
· Read about a new program to help families share FH information
· Answer written response and multiple-choice questions about these resources

It should take you about 25 minutes to review the resources and complete the survey. We ask that you be as detailed as possible when answering the written response questions.

At the end of the survey, you will be invited to share a similar survey with your blood relatives and your spouse or partner.

Choosing not to take the survey will have no effect on your current medical care. If you choose to take the survey, you may exit it at any time.

Click the "Next" button at the bottom of the screen to start the survey.

If you have questions, concerns, or problems with the survey, please call our team at 1-866-910-6486, Option 2. You may also email us at IMPACTFH@geisinger.edu.

Thank you, we sincerely value your input.

IMPACT-FH: FH Foundation Survey for Individuals with FH

Demographic Information

Please answer the following questions.

* 1. Please type your age:

* 2. What is your biological sex?

- Male
- Female
- Prefer not to say

* 3. What is your annual household income?

- < $25,000
- $25 – 50,000
- $50 – 75,000
- $75 – 100,000
- > $100,000
- Prefer not to answer

* 4. What is your highest level of school completed?

- Some High School
- Graduated High School/GED
- Some College
- Associate’s Degree
- Bachelor’s Degree
- Graduate/Professional Degree
- Prefer not to answer

* 5. What state do you currently live in?

State/Province -- select state --

* 6. How did you find out that you have FH? *(Check all that apply)*

- [ ] Learned of FH through genetic testing results
- [ ] Diagnosed by a doctor or other medical professional based on my cholesterol results and personal/family history
- [ ] A family member told me about their FH diagnosis, and I told a doctor/did cholesterol testing
- [ ] Did my own research based on my cholesterol results and personal/family history and sought out an FH diagnosis
- [ ] Other, please explain:

* 7. Have you had genetic testing since your FH diagnosis?

- ○ Yes
- ○ No
- ○ Genetic testing was how I first received my diagnosis of FH

Demographic Information - Genetic Testing Results

Please answer the following question.

* 8. What was the result of your genetic test?

- ◯ Positive result
- ◯ Negative result
- ◯ Variant of Unknown Significance

Demographic Information - FH Diagnosis

Please answer the following question.

* 9. Were you the first person in your family to be diagnosed with FH?

- ◯ Yes
- ◯ No

IMPACT-FH: FH Foundation Survey for Individuals with FH

Demographic Information - FH Diagnosis Age

Please answer the following question.

* 10. Please type in the age when you were first diagnosed with FH:

IMPACT-FH: FH Foundation Survey for Individuals with FH

Demographic Information - FH Diagnosis Year

Please answer the following question.

* 11. When was the first person in your family diagnosed with FH? Please type year or your best estimation:

IMPACT-FH: FH Foundation Survey for Individuals with FH

FH Infographic

Please review the infographic below, then click "Next" at the bottom of the page to continue the survey.

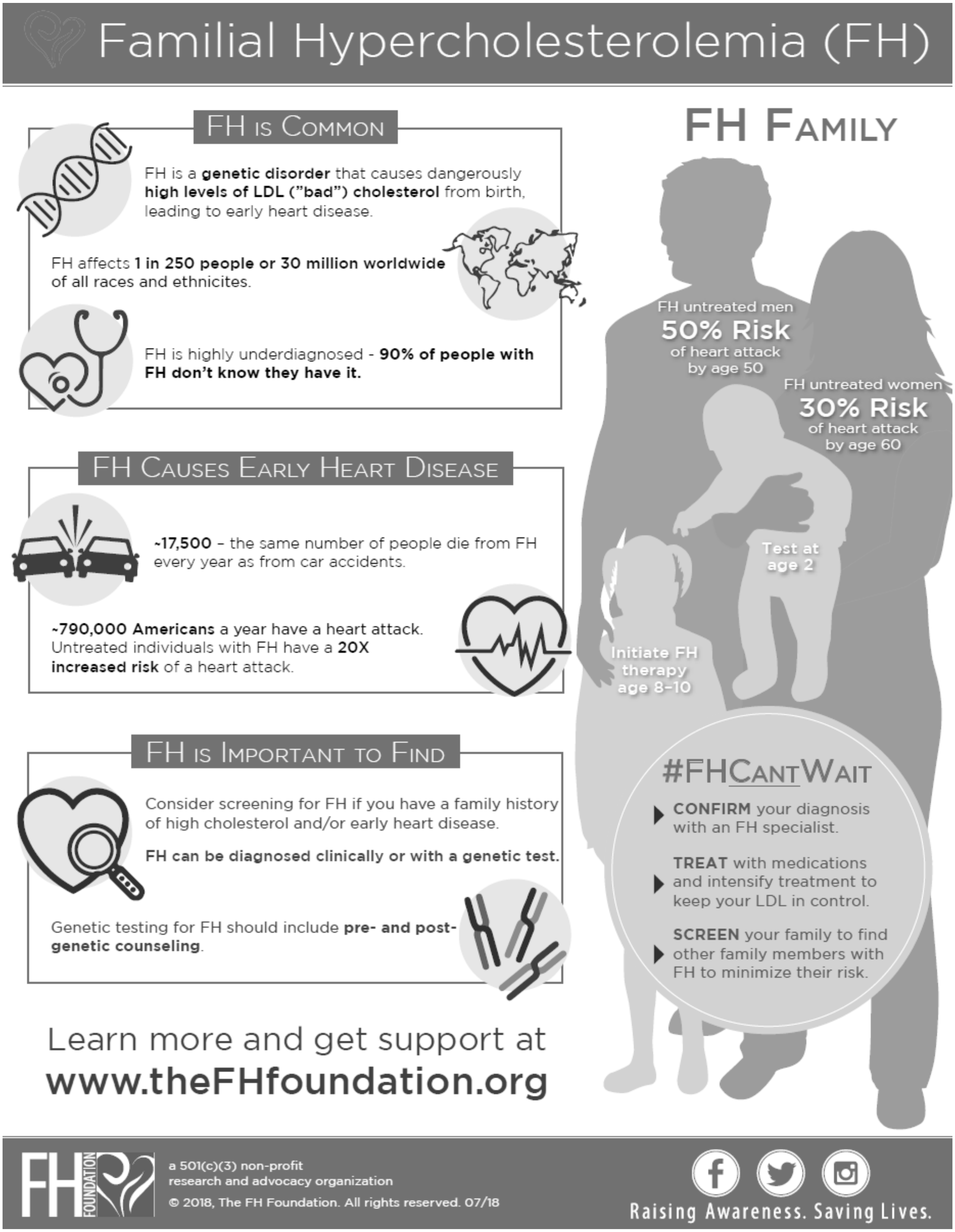

Familial Hypercholesterolemia (FH)
FH is Common
FH is a genetic disorder that causes dangerously high levels of LDL ("bad") cholesterol from birth, leading to early heart disease.
FH affects 1 in 250 people or 30 million worldwide of all races and ethnicites.
FH is highly underdiagnosed - 90% of people with FH don't know they have it.
FH Causes Early Heart Disease
~17,500 – the same number of people die from FH every year as from car accidents.
~790,000 Americans a year have a heart attack. Untreated individuals with FH have a 20X increased risk of a heart attack.
FH is Important to Find
Consider screening for FH if you have a family history of high cholesterol and/or early heart disease.
FH can be diagnosed clinically or with a genetic test.
Genetic testing for FH should include pre- and post-genetic counseling.
Learn more and get support at
www.theFHfoundation.org
FH Family
FH untreated men
50% Risk
of heart attack
by age 50
FH untreated women
30% Risk
of heart attack
by age 60
Test at age 2
Initiate FH therapy age 8–10
#FHCantWait
CONFIRM your diagnosis with an FH specialist.
TREAT with medications and intensify treatment to keep your LDL in control.
SCREEN your family to find other family members with FH to minimize their risk.
FH Foundation
a 501(c)(3) non-profit
research and advocacy organization
© 2018, The FH Foundation. All rights reserved. 07/18
Raising Awareness. Saving Lives.

IMPACT-FH: FH Foundation Survey for Individuals with FH

Section 1: Dear Family Letter

The Dear Family Letter is a resource that was developed at Geisinger. It is given to people who receive a genetic diagnosis of FH through Geisinger's MyCode Community Health Initiative to help share their result with relatives of their choosing.

Please read the sample Dear Family Letter below before moving on to the next page.

You may return to this page at any time to review the Dear Family Letter by using the "Back" button at the bottom of the page. Your responses will be saved if you go back to review the letter.

6/17/2020

Dear Ms. Jane Doe,

I learned that my *LDLR* gene does not work correctly through the Geisinger MyCode study. This gene change puts me at higher risk for early heart attack and stroke from inherited high cholesterol. Another name for this genetic risk is Familial Hypercholesterolemia (FH).

Gene changes run in families. Parents, children, brothers and sisters of people with a *LDLR* gene change have a 50% chance of having the same gene change and health risks. Other family members (aunts, uncles, nieces, nephews, cousins, grandchildren) may also have the same gene change and health risks.

People who have FH often need extra medical care. This extra care may include looking for and treating potentially life-threatening heart problems.

A simple "yes/no" blood or saliva test can tell you if you also have the same gene change and health risks.

You may be able to get this "yes/no" test for free or at lower-cost from the same lab that ran my test. Free genetic testing for family members is offered for 90 days after **6/10/2020**, my report date. The appointment with the provider to order this test will be billed to you or your insurance.

What you should do next:
- **Call the Geisinger MyCode Genomic Screening and Counseling team (toll-free) at 1-844-250-8031.** They can schedule an appointment to start the "yes/no" testing process. They can also answer your questions.
- **If you do not live in PA, you can find a genetic counselor to order this test and discuss your risks at:** https://www.nsgc.org/page/find-a-genetic-counselor

The healthcare provider ordering your test will need this information:
c.2054C>T p.Pro685Leu in the *LDLR* gene (NM_000527.5)
Laboratory: Invitae.

This letter gives my permission for Geisinger to share my genetic test results with you for your care. **Please bring this letter with you to your appointment.**

If you have any questions or concerns, please call the Geisinger MyCode team (toll-free) at **1-844-250-8031**.

Sincerely,

____
Mr. John Doe

INVITAE

SECONDARY FINDINGS SCREEN RESULTS

**Patient name:**	John Doe	**Sample type:**	gDNA	**Report date:**	
**DOB:**		**Sample collection date:**		**Invitae #:**	
**Sex:**	Male	**Sample accession date:**		**Clinical team:**	
**MRN:**					

Test performed

Sequence analysis and deletion/duplication testing of the 59 genes listed in the Genes Analyzed section.

- Secondary Findings Add-on

RESULT: POSITIVE

A clinically significant genetic change was found in the LDLR gene, which is associated with a heart-related condition.

GENE	VARIANT	ZYGOSITY	VARIANT CLASSIFICATION
LDLR	c.2054C>T (p.Pro685Leu)	heterozygous	PATHOGENIC

About this test

This test evaluates 59 genes for variants (genetic changes) that indicate a significantly increased risk of developing certain types of cancer, heart-related conditions, or other types of actionable medical genetic conditions. These are disorders for which effective medical interventions and preventive measures are known and available. Genetic changes of uncertain significance are not included in this report; however, if additional evidence becomes available to indicate that a previously uncertain genetic change is clinically significant, Invitae will update this report and provide notification.

Next steps

- This is a medically important result that should be discussed with an appropriate healthcare provider. Genetic counseling is recommended to discuss the implications of this result and potential next steps.
- Consider sharing this result with relatives as they may also be at risk. Details on our Family Variant Testing program can be found at www.invitae.com/family.
- Register your test at www.invitae.com/patients to download a digital copy of your results. You can also access educational resources about how your results can help inform your health.

Clinical Summary

A Pathogenic variant, c.2054C>T (p.Pro685Leu), was identified in LDLR.

Laboratory Director Tina Hambuch, Ph.D., FACMG
NY Laboratory Director Swaroop Aradhya, Ph.D., FACMG
Invitae 1400 16th Street, San Francisco, CA 94103
E: clientservices@invitae.com P: 415.374.7782 or 800.436.3037

IMPACT-FH: FH Foundation Survey for Individuals with FH

Section 1: Dear Family Letter

Please answer the following questions.

You may use the "Back" button at the bottom of the page to return to the sample Dear Family Letter. Your responses will be saved if you go back to review the letter.

* 12. Do you consider this letter to be a reliable/trustworthy source for medical information?

- ○ Yes
- ○ No
- ○ I'm not sure
- ○ Other, please explain:

* 13. How easy is it to understand the information in the letter?

- ○ Very easy
- ○ Somewhat easy
- ○ Neutral
- ○ Somewhat difficult
- ○ Very difficult

* 14. How should we make this letter available to you to share with your relatives? *(Check all that apply)*

- ☐ Share it to my email
- ☐ Share it to my patient portal (e.g., MyChart, MyGeisinger, etc.)
- ☐ Mail paper copies to me
- ☐ Other, please explain:

* 15. Would you send this type of letter to a relative?

- Yes
- No

IMPACT-FH: FH Foundation Survey for Individuals with FH

Section 1: Dear Family Letter

Please be detailed in your responses to the below question, so we can better understand how to improve our resources for individuals and families with FH.

You may use the "Back" button at the bottom of the page to return to the sample Dear Family Letter. Your responses will be saved if you go back to review the letter.

* 16. Why would you NOT send this letter to relatives?

IMPACT-FH: FH Foundation Survey for Individuals with FH

Section 1: Dear Family Letter

Please answer the following questions.

You may use the "Back" button at the bottom of the page to return to the sample Dear Family Letter. Your responses will be saved if you go back to review the letter.

* 17. Who would you send this type of letter to? *(Check all that apply)*

- ☐ Mother
- ☐ Father
- ☐ Sister(s)
- ☐ Brother(s)
- ☐ Son(s)
- ☐ Daughter(s)
- ☐ Grandchild(ren)
- ☐ Grandparents – mother's side
- ☐ Grandparents – father's side
- ☐ Cousin(s) – mother's side
- ☐ Cousin(s) – father's side
- ☐ Aunt(s) – mother's side
- ☐ Aunt(s) – father's side
- ☐ Uncle(s) – mother's side
- ☐ Uncle(s) – father's side
- ☐ Other, please specify:

* 18. Why would you send the letter to these people? *Please be detailed in your response.*

IMPACT-FH: FH Foundation Survey for Individuals with FH

Section 1: Dear Family Letter

Please be detailed in your responses to the below questions, so we can better understand how to improve our resources for individuals and families with FH.

You may use the "Back" button at the bottom of the page to return to the sample Dear Family Letter. Your responses will be saved if you go back to review the letter.

* 19. What other information do you think could be included in the letter to help your relatives take next steps to find out if they have FH?

20. What else do you want to share with us about the letter and/or how to make it better?

IMPACT-FH: FH Foundation Survey for Individuals with FH

Section 2: Chatbot

A chatbot is an online conversational tool. People with FH are offered a chatbot to help them more easily share information about FH with relatives. Relatives of the individual’s choosing are sent the chatbot to help them learn more about FH and their risks.

Please watch the chatbot video below before moving on to the next page.

If you are having trouble playing this video within the survey, please copy and paste the following link *in a new tab or window*: https://youtu.be/aTDKdodrIzs

You may return to this page at any time to re-watch the chatbot video by using the "Back" button at the bottom of the page. Your responses will be saved if you go back to re-watch the video.

IMPACT-FH: FH Foundation Survey for Individuals with FH

Section 2: Chatbot

Please answer the following question.

You may use the "Back" button at the bottom of the page to return to the chatbot video. Your responses will be saved if you go back to re-watch the video.

* 21. Would you send this type of chatbot link to any of your relatives?

- Yes
- No

IMPACT-FH: FH Foundation Survey for Individuals with FH

Section 2: Chatbot

Please answer the following question.

You may use the "Back" button at the bottom of the page to return to the chatbot video. Your responses will be saved if you go back to re-watch the video.

* 22. Why would you NOT send the chatbot to any of your relatives? *(Check all that apply)*

- [ ] I am not comfortable with technology
- [ ] My relatives are not comfortable with technology
- [ ] I would prefer to call or tell them myself
- [ ] Other, please explain:

IMPACT-FH: FH Foundation Survey for Individuals with FH

Section 3: Chatbot

Please answer the following questions.

You may use the "Back" button at the bottom of the page to return to the chatbot video. Your responses will be saved if you go back to re-watch the video.

* 23. Who would you send the chatbot link to? *(Check all that apply)*

- [ ] Mother
- [ ] Father
- [ ] Sister(s)
- [ ] Brother(s)
- [ ] Son(s)
- [ ] Daughter(s)
- [ ] Grandchild(ren)
- [ ] Grandparents – mother’s side
- [ ] Grandparents – father’s side
- [ ] Cousin(s) – mother’s side
- [ ] Cousin(s) – father’s side
- [ ] Aunt(s) – mother's side
- [ ] Aunt(s) – father's side
- [ ] Uncle(s) – mother’s side
- [ ] Uncle(s) – father’s side
- [ ] Other, please specify:

* 24. Why would you send the chatbot to these people? *Please be detailed in your response.*

Section 2: Chatbot

Please answer the following question.

You may use the "Back" button at the bottom of the page to return to the chatbot video. Your responses will be saved if you go back to re-watch the video.

* 25. When would you send the chatbot link to relatives?

- ◯ Call/talk first, then send the link
- ◯ Send the link immediately
- ◯ Some I'd call/talk to first, some I'd just send the link
- ◯ Other

IMPACT-FH: FH Foundation Survey for Individuals with FH

Section 2: Chatbot

Please answer the following question.

You may use the "Back" button at the bottom of the page to return to the chatbot video. Your responses will be saved if you go back to re-watch the video.

* 26. Please explain when you would send the chatbot link to family. *Please be detailed in your response.*

IMPACT-FH: FH Foundation Survey for Individuals with FH

Section 2: Chatbot

Please answer the following questions.

You may use the "Back" button at the bottom of the page to return to the chatbot video. Your responses will be saved if you go back to re-watch the video.

* 27. Would you wait to send the chatbot until after you spoke with a doctor or a genetic counselor?

- ○ Yes
- ○ No
- ○ I'm not sure
- ○ Other, please explain:

* 28. Select the options you feel comfortable using to share the chatbot. *(Check all that apply)*

- ☐ Text message
- ☐ Email
- ☐ Facebook messenger
- ☐ Other, please specify:

* 29. What would influence how you share the chatbot with each relative? *(Check all that apply)*

- [ ] How well I know the relative
- [ ] How closely related we are
- [ ] Contact information I have for relatives
- [ ] How I think my relative would like to receive this information
- [ ] Things I know about my relative's mental health
- [ ] Things I know about my relative's physical health
- [ ] My relative's attitude toward health/healthcare
- [ ] My relative's comfort level with technology
- [ ] Other, please explain:

* 30. Do you consider the chatbot to be a reliable/trustworthy source for medical information?

- ○ Yes
- ○ No
- ○ I'm not sure
- ○ Other, please specify:

* 31. How easy is it to understand the information in the chatbot?

- ○ Very easy
- ○ Somewhat easy
- ○ Neutral
- ○ Somewhat difficult
- ○ Very difficult

Section 2: Chatbot

Please answer the following questions.

You may use the "Back" button at the bottom of the page to return to the chatbot video. Your responses will be saved if you go back to re-watch the video.

* 32. What can we improve about the chatbot to make it more likely you would share the chatbot with your relatives?

* 33. What can we improve about the chatbot to make it more likely your relatives will take action?

34. What else do you want to share with us about the chatbot and/or how to make it better?

IMPACT-FH: FH Foundation Survey for Individuals with FH

Section 3: Direct Contact Program

The Direct Contact Program is a new program being designed by the FH Foundation and Geisinger. It will be another way for individuals diagnosed with FH to share their diagnosis with the relatives of their choosing. The Direct Contact Program has not been finalized yet. The description below is a general idea of what the program will look like.

Please read the information below before moving on to the next page.

You may return to this page at any time to review this information by using the "Back" button at the bottom of the page. Your responses will be saved if you go back to review this information.

What is a Direct Contact Program?

Step 1

A person gets tested, via genetic testing and/or cholesterol testing, and finds out they have Familial Hypercholesterolemia (FH).

Step 2

The person with FH gives a healthcare provider permission to share their FH diagnosis with their at-risk relatives.

- The person with FH can pick which at-risk relatives they want the healthcare provider to contact and gives the healthcare provider contact information (for example, address, email, telephone number) for each of those relatives.

Step 3

The healthcare provider contacts at-risk relatives to share that their family member has FH and to explain their risks of heart disease if they have FH, too.

Step 4

A healthcare provider, like a genetic counselor or doctor, can counsel relatives and help them get testing.

Why would someone use a Direct Contact Program?

Sharing complex health information about FH with at-risk relatives can be hard.

Talking to a healthcare provider can motivate at-risk relatives to get tested for FH.

If relatives test positive for FH, they can get care for their FH health risk sooner.

FH FOUNDATION | Geisinger

IMPACT-FH: FH Foundation Survey for Individuals with FH

Section 3: Direct Contact Program

Please answer the following questions.

You may use the "Back" button at the bottom of the page to return to the information about the Direct Contact Program. Your responses will be saved if you go back to review the information.

* 35. Imagine if your doctor's office offered a Direct Contact Program. In this program, you would provide the name and contact information of some, or all, of your relatives at risk for FH. With your permission, a healthcare provider from the program would contact your relatives to tell them that “A family member has received an FH diagnosis and you could be at risk too.”

Using a 1-5 scale, where 1 means Not at All Likely and 5 means Extremely Likely, indicate how likely you would be to use such a Direct Contact Program.

1. Not at All Likely	2. Somewhat Unlikely	3. Neutral	4. Somewhat Likely	5. Extremely Likely
○	○	○	○	○

* 36. If you used this Direct Contact Program to share information about FH with your at-risk relatives, would you want the provider to share your name and your FH result?

○ Yes

○ No

○ Unsure

○ Depends on the relative

* 37. How helpful would it be if a healthcare provider offered to contact your relatives on your behalf to inform them of your FH diagnosis and their potential risk and need for screening?

- ◯ Very helpful
- ◯ Somewhat helpful
- ◯ Neutral
- ◯ Somewhat unhelpful
- ◯ Not helpful at all
- ◯ Unsure
- ◯ Other, please specify:

* 38. What type of healthcare provider would you feel comfortable directly contacting your relatives? *(Check all that apply)*

- ☐ Genetic counselor (a healthcare provider with expertise in genetics)
- ☐ Nurse
- ☐ Primary care provider
- ☐ Specialist (e.g., cardiologist, lipidologists, etc.)
- ☐ Pharmacist
- ☐ Medical staff trained for this purpose
- ☐ Other, please specify:

* 39. Who would you want the Direct Contact Program to contact on your behalf?

- ◯ All my at-risk relatives
- ◯ Only my at-risk relatives of my choosing
- ◯ None of my at-risk relatives

IMPACT-FH: FH Foundation Survey for Individuals with FH

Section 3: Direct Contact Program

Please answer the following question.

You may use the "Back" button at the bottom of the page to return to the information about the Direct Contact Program. Your responses will be saved if you go back to review the information.

40. How would you decide which relatives to use Direct Contact for? *(Check all that apply)*

- [ ] How well I know the relative
- [ ] Contact information I have for relative
- [ ] How I think my relative would like to receive this information
- [ ] Things I know about my relative's mental health
- [ ] Things I know about my relative's physical health
- [ ] How closely related we are
- [ ] My relative's attitude toward health/healthcare
- [ ] Other, please explain:

IMPACT-FH: FH Foundation Survey for Individuals with FH

Section 3: Direct Contact Program

Please answer the following questions.

You may use the "Back" button at the bottom of the page to return to the information about the Direct Contact Program. Your responses will be saved if you go back to review the information.

* 41. Think about the at-risk relatives you would want the Direct Contact Program to contact. What contact information for your relatives would you be comfortable providing? *(Check all that apply)*

- ☐ Phone number
- ☐ Mailing address
- ☐ Email address
- ☐ Other, please explain:

* 42. How acceptable do you think it would be to your at-risk relatives to receive information about your FH diagnosis directly from an FH expert in the Direct Contact Program?

- ○ It would be very acceptable to most of my relatives
- ○ Most would accept it, but a few would not accept it
- ○ In general, my relatives would find it neither acceptable nor unacceptable
- ○ A few would accept it, but most would not accept it
- ○ None of my relatives would accept it
- ○ Unsure

FH FOUNDATION | Geisinger

IMPACT-FH: FH Foundation Survey for Individuals with FH

Section 3: Direct Contact Program

Please be detailed in your responses to the below questions, so we can better understand how to improve our resources for individuals and families with FH.

You may use the "Back" button at the bottom of the page to return to the information about the Direct Contact Program. Your responses will be saved if you go back to review the information.

* 43. Ideally, how would you want a healthcare provider to help you share information with relatives about their FH risks?

44. What else do you want to share with us about the Direct Contact Program and/or how to make it better?

* 45. If you did not want to use the letter, chatbot, or the Direct Contact Program to share your FH result with your at-risk relatives, how would you inform these family members?

Additional Opportunities

* 46. Would you be interested in taking part in further opportunities on how healthcare providers can support families talking about FH?

- ◯ Yes
- ◯ No

IMPACT-FH: FH Foundation Survey for Individuals with FH

Additional Opportunities - Contact Information

* 47. Please provide your name, phone number and email address so we can contact you with more information on ways to take part in more FH projects:

Name

Email Address

Phone Number

Share a Survey with your Family Members!

We are also interested in understanding how *your family members* feel about these materials.

If you are willing to share a similar survey with your blood relatives, or your spouse/partner, *please copy the link below and share it with them*.

You may share the link with your family members in any way you choose.

Family Member Survey Link: [Link to Corresponding Family Member Survey]

Supplementary File 4. Family Member Survey

IMPACT-FH: Family Member Survey

Welcome and Instructions

Greetings!

Geisinger, a health system in Pennsylvania, and the FH Foundation, a non-profit research and advocacy organization, want to learn how to better support people as they share information about Familial Hypercholesterolemia (FH) with their relatives.

Your responses will help us improve our current resources and design new ways for people to talk about FH with their family.

During the survey, you will be asked to:

- **Review a letter**
- **Watch a video of a chatbot**
- **Read about a new program to help families share FH information**
- **Answer written response and multiple-choice questions about these resources**

It should take you about 25 minutes to review the resources and complete the survey. We ask that you be as detailed as possible when answering the written response questions.

At the end of the survey, you will be invited to share this survey with your blood relatives and your spouse or partner.

Choosing not to take the survey will have no effect on your current medical care. If you choose to take the survey, you may exit it at any time.

Click the "Next" button at the bottom of the screen to start the survey.

If you have questions, concerns, or problems with the survey, please call our team at 1-866-910-6486, Option 2. You may also email us at IMPACTFH@geisinger.edu.

Thank you, we sincerely value your input.

FH FOUNDATION | Geisinger

IMPACT-FH: Family Member Survey

Demographic Information

Please answer the following questions.

* 1. How did you receive this survey?

- ◯ My family member, who is a Geisinger patient, sent me the survey
- ◯ My family member sent me the survey
- ◯ The FH Foundation sent me the survey
- ◯ The FH Foundation's social media page

* 2. Please type your age:

[ ]

* 3. What is your biological sex?

- ◯ Male
- ◯ Female
- ◯ Prefer not to say

* 4. What is your annual household income?

- ◯ < $25,000
- ◯ $25 – 50,000
- ◯ $50 – 75,000
- ◯ $75 – 100,000
- ◯ > $100,000
- ◯ Prefer not to answer

* 5. What is your highest level of school completed?

- ◯ Some High School
- ◯ Graduated High School/GED
- ◯ Some College
- ◯ Associate’s Degree
- ◯ Bachelor’s Degree
- ◯ Graduate/Professional Degree
- ◯ Prefer not to answer

* 6. What state do you currently live in?

State/Province [-- select state --]

* 7. What is your relationship to the first person diagnosed with FH in your family? Please type:

If you were the first person diagnosed with FH in your family, please write "I was the first person."

[ ]

* 8. Have you received testing for FH?

- ◯ Yes, doctor tested my cholesterol
- ◯ Yes, genetic testing
- ◯ No
- ◯ Unsure

IMPACT-FH: Family Member Survey

Demographic Information - FH Testing

Please answer the following questions.

* 9. Please type how old you were when you got tested for FH:

* 10. What was the result of the test?

- I have FH
- I do not have FH
- Unsure

IMPACT-FH: Family Member Survey

FH Infographic

Please review the infographic below to learn a little more about FH. This will help you understand questions on the survey. Click "Next" at the bottom of the page to proceed.

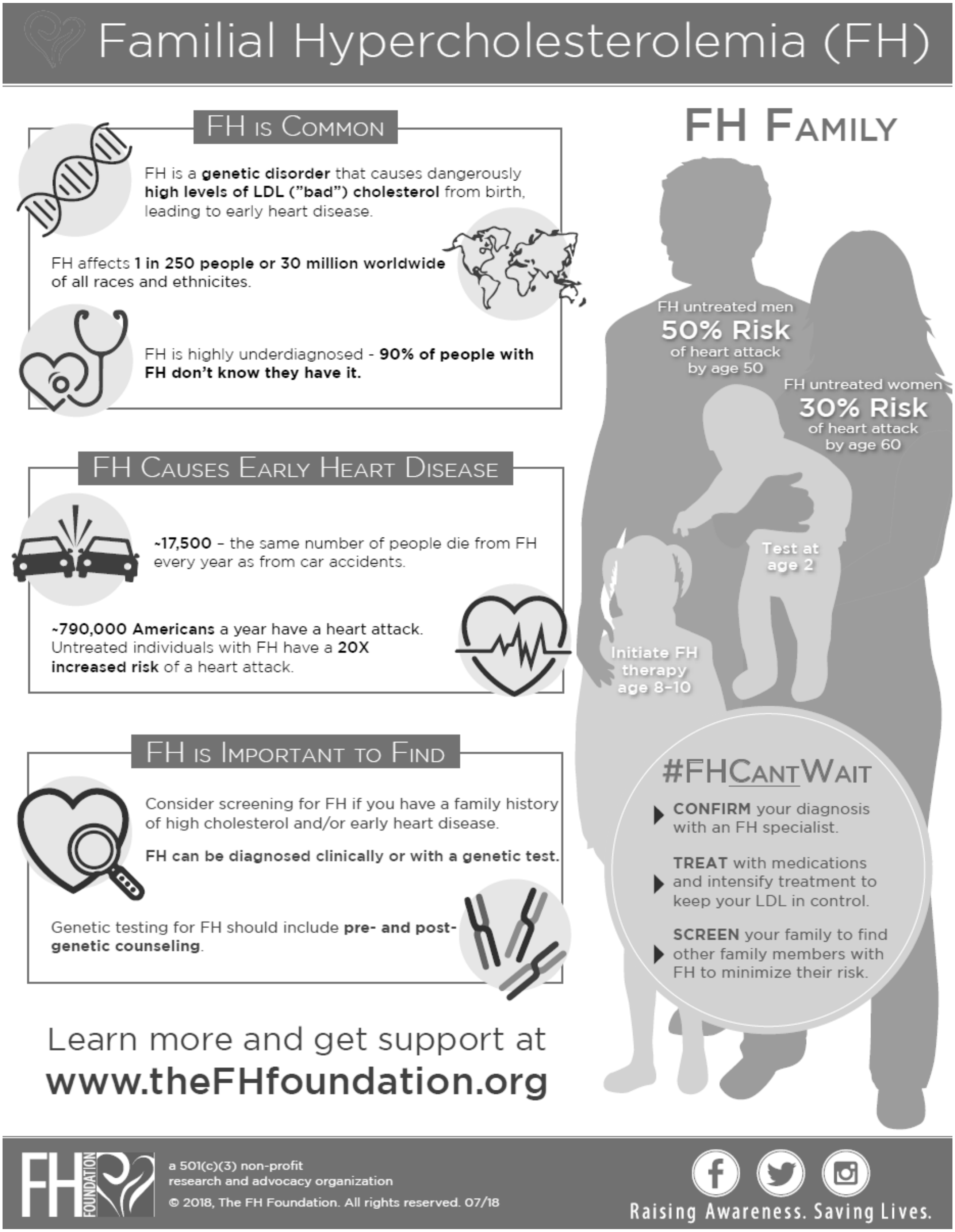
Familial Hypercholesterolemia (FH)
FH is Common
FH is a genetic disorder that causes dangerously high levels of LDL ("bad") cholesterol from birth, leading to early heart disease.
FH affects 1 in 250 people or 30 million worldwide of all races and ethnicites.
FH is highly underdiagnosed - 90% of people with FH don't know they have it.
FH Causes Early Heart Disease
~17,500 – the same number of people die from FH every year as from car accidents.
~790,000 Americans a year have a heart attack. Untreated individuals with FH have a 20X increased risk of a heart attack.
FH is Important to Find
Consider screening for FH if you have a family history of high cholesterol and/or early heart disease.
FH can be diagnosed clinically or with a genetic test.
Genetic testing for FH should include pre- and post-genetic counseling.
Learn more and get support at
www.theFHfoundation.org
FH Family
FH untreated men
50% Risk
of heart attack by age 50
FH untreated women
30% Risk
of heart attack by age 60
Test at age 2
Initiate FH therapy age 8–10
#FHCantWait
CONFIRM your diagnosis with an FH specialist.
TREAT with medications and intensify treatment to keep your LDL in control.
SCREEN your family to find other family members with FH to minimize their risk.
FH FOUNDATION
a 501(c)(3) non-profit research and advocacy organization
© 2018, The FH Foundation. All rights reserved. 07/18
Raising Awareness. Saving Lives.

IMPACT-FH: Family Member Survey

Section 1: Dear Family Letter

The Dear Family Letter is a resource that was developed at Geisinger. It is given to people who receive a genetic diagnosis of FH through Geisinger's MyCode Community Health Initiative to help share their result with relatives of their choosing.

Please read the sample Dear Family Letter below before moving on to the next page.

You may return to this page at any time to review the Dear Family Letter by using the "Back" button at the bottom of the page. Your responses will be saved if you go back to review the letter.

mycode Geisinger

6/17/2020

Dear Ms. Jane Doe,

I learned that my *LDLR* gene does not work correctly through the Geisinger MyCode study. This gene change puts me at higher risk for early heart attack and stroke from inherited high cholesterol. Another name for this genetic risk is Familial Hypercholesterolemia (FH).

Gene changes run in families. Parents, children, brothers and sisters of people with a *LDLR* gene change have a 50% chance of having the same gene change and health risks. Other family members (aunts, uncles, nieces, nephews, cousins, grandchildren) may also have the same gene change and health risks.

People who have FH often need extra medical care. This extra care may include looking for and treating potentially life-threatening heart problems.

A simple “yes/no” blood or saliva test can tell you if you also have the same gene change and health risks.

You may be able to get this “yes/no” test for free or at lower-cost from the same lab that ran my test. Free genetic testing for family members is offered for 90 days after **6/10/2020**, my report date. The appointment with the provider to order this test will be billed to you or your insurance.

What you should do next:

- **Call the Geisinger MyCode Genomic Screening and Counseling team (toll-free) at 1-844-250-8031.** They can schedule an appointment to start the “yes/no” testing process. They can also answer your questions.

- **If you do not live in PA, you can find a genetic counselor to order this test and discuss your risks at:** https://www.nsgc.org/page/find-a-genetic-counselor

The healthcare provider ordering your test will need this information:

c.2054C>T p.Pro685Leu in the *LDLR* gene (NM_000527.5)
Laboratory: Invitae.

This letter gives my permission for Geisinger to share my genetic test results with you for your care. **Please bring this letter with you to your appointment.**

If you have any questions or concerns, please call the Geisinger MyCode team (toll-free) at **1-844-250-8031**.

Sincerely,

____

Mr. John Doe

INVITAE

SECONDARY FINDINGS SCREEN RESULTS

**Patient name:** John Doe	**Sample type:** gDNA	**Report date:**
**DOB:**	**Sample collection date:**	**Invitae #:**
**Sex:** Male	**Sample accession date:**	**Clinical team:**
**MRN:**		

Test performed

Sequence analysis and deletion/duplication testing of the 59 genes listed in the Genes Analyzed section.

- Secondary Findings Add-on

RESULT: POSITIVE

A clinically significant genetic change was found in the LDLR gene, which is associated with a heart-related condition.

GENE	VARIANT	ZYGOSITY	VARIANT CLASSIFICATION
LDLR	c.2054C>T (p.Pro685Leu)	heterozygous	PATHOGENIC

About this test

This test evaluates 59 genes for variants (genetic changes) that indicate a significantly increased risk of developing certain types of cancer, heart-related conditions, or other types of actionable medical genetic conditions. These are disorders for which effective medical interventions and preventive measures are known and available. Genetic changes of uncertain significance are not included in this report; however, if additional evidence becomes available to indicate that a previously uncertain genetic change is clinically significant, Invitae will update this report and provide notification.

Next steps

- This is a medically important result that should be discussed with an appropriate healthcare provider. Genetic counseling is recommended to discuss the implications of this result and potential next steps.
- Consider sharing this result with relatives as they may also be at risk. Details on our Family Variant Testing program can be found at www.invitae.com/family.
- Register your test at www.invitae.com/patients to download a digital copy of your results. You can also access educational resources about how your results can help inform your health.

Clinical Summary

A Pathogenic variant, c.2054C>T (p.Pro685Leu), was identified in LDLR.

Laboratory Director Tina Hambuch, Ph.D., FACMG
NY Laboratory Director Swaroop Aradhya, Ph.D., FACMG
Invitae 1400 16th Street, San Francisco, CA 94103
E: clientservices@invitae.com P: 415.374.7782 or 800.436.3037

IMPACT-FH: Family Member Survey

Section 1: Dear Family Letter

Please answer the following question.

You may use the "Back" button at the bottom of the page to return to the sample Dear Family Letter. Your responses will be saved if you go back to review the letter.

* 11. Would you want to receive a letter like this from a relative who has FH?

- ◯ Yes
- ◯ No

IMPACT-FH: Family Member Survey

Section 1: Dear Family Letter

Please answer the following questions.

You may use the "Back" button at the bottom of the page to return to the sample Dear Family Letter. Your responses will be saved if you go back to review the letter.

* 12. How would you prefer to receive a letter like this from a relative who has FH? *(Check all that apply)*

- ☐ Mail it to me
- ☐ Email it to me
- ☐ Have a conversation with my relative using content from the letter
- ☐ Other, please explain:

* 13. Do you consider this letter to be a reliable/trustworthy source for medical information?

- ○ Yes
- ○ No
- ○ I'm not sure
- ○ Other, please explain:

FH FOUNDATION | Geisinger

IMPACT-FH: Family Member Survey

Section 1: Dear Family Letter

Please be detailed in your responses to the below questions, so we can better understand how to improve our resources for individuals and families with FH.

You may use the "Back" button at the bottom of the page to return to the sample Dear Family Letter. Your responses will be saved if you go back to review the letter.

* 14. What would you do if you received this letter from your relatives?

* 15. What other information would you want from a letter like this?

* 16. What can we do to improve this letter to help you or your relatives who may have FH take next steps to find out if you or they have FH?

17. What else do you want to share with us about the letter and/or how to make it better?

IMPACT-FH: Family Member Survey

Section 2: Chatbot

A chatbot is an online conversational tool. People with FH are offered a chatbot to help them more easily share information about FH with relatives. Relatives of the individual's choosing are sent the chatbot to help them learn more about FH and their risks.

Please watch the chatbot video below before moving on to the next page.

If you are having trouble playing this video within the survey, please copy and paste the following link *in a new tab or window*: https://youtu.be/aTDKdodrIzs

You may return to this page at any time to re-watch the chatbot video by using the "Back" button at the bottom of the page. Your responses will be saved if you go back to re-watch the video.

IMPACT-FH: Family Member Survey

Section 2: Chatbot

Please answer the following question.

You may use the "Back" button at the bottom of the page to return to the chatbot video. Your responses will be saved if you go back to re-watch the video.

* 18. If your relative sent you the chatbot, would you use it?

- ◯ Yes
- ◯ No
- ◯ I'm not sure
- ◯ Other, please specify:

IMPACT-FH: Family Member Survey

Section 2: Chatbot

Please answer the following question.

You may use the "Back" button at the bottom of the page to return to the chatbot video. Your responses will be saved if you go back to re-watch the video.

* 19. Why would you use the chatbot if a relative sent it to you? *Please be detailed in your response.*

IMPACT-FH: Family Member Survey

Section 2: Chatbot

Please answer the following question.

You may use the "Back" button at the bottom of the page to return to the chatbot video. Your responses will be saved if you go back to re-watch the video.

* 20. Why would you NOT use the chatbot if a relative sent it to you? *Please be detailed in your response.*

IMPACT-FH: Family Member Survey

Section 2: Chatbot

Please answer the following question.

You may use the "Back" button at the bottom of the page to return to the chatbot video. Your responses will be saved if you go back to re-watch the video.

* 21. Why are you unsure about whether you would use the chatbot if a relative sent it to you? *Please be detailed in your response.*

IMPACT-FH: Family Member Survey

Section 2: Chatbot

Please answer the following questions.

You may use the "Back" button at the bottom of the page to return to the chatbot video. Your responses will be saved if you go back to re-watch the video.

* 22. Would you want reminders or check-ins from a chatbot to remind you to talk with your doctor/get tested?

- ○ Yes
- ○ No
- ○ I'm not sure
- ○ Other, please explain:

* 23. What would you feel comfortable using the chatbot for? *(Check all that apply)*

- ☐ Ordering a mail kit for genetic testing
- ☐ Finding a genetic counselor or doctor near you
- ☐ Watching a video on FH risks
- ☐ Connecting to the FH Foundation for support
- ☐ Finding information on FH
- ☐ Other, please explain:

* 24. Do you consider the chatbot to be a reliable/trustworthy source for medical information?

- ◯ Yes
- ◯ No
- ◯ I'm not sure
- ◯ Other, please specify:

IMPACT-FH: Family Member Survey

Section 2: Chatbot

Please be detailed in your responses to the below questions, so we can better understand how to improve our resources for individuals and families with FH.

You may use the "Back" button at the bottom of the page to return to the chatbot video. Your responses will be saved if you go back to re-watch the video.

* 25. What can we improve about the chatbot to help you or your relatives who may have FH take next steps to find out if you or they have FH?

26. What else do you want to share with us about the chatbot and/or how to make it better?

IMPACT-FH: Family Member Survey

Section 3: Direct Contact Program

The Direct Contact Program is a new program being designed by Geisinger and the FH Foundation. It will be another way for individuals diagnosed with FH to share their diagnosis with the relatives of their choosing. The Direct Contact Program has not been finalized yet. The description below is a general idea of what the program will look like.

Please read the information below before moving on to the next page.

You may return to this page at any time to review this information by using the "Back" button at the bottom of the page. Your responses will be saved if you go back to review this information.

What is a Direct Contact Program?

Step 1

A person gets tested, via genetic testing and/or cholesterol testing, and finds out they have Familial Hypercholesterolemia (FH).

Step 2

The person with FH gives a healthcare provider permission to share their FH diagnosis with their at-risk relatives.

- The person with FH can pick which at-risk relatives they want the healthcare provider to contact and gives the healthcare provider contact information (for example, address, email, telephone number) for each of those relatives.

Step 3

The healthcare provider contacts at-risk relatives to share that their family member has FH and to explain their risks of heart disease if they have FH, too.

Step 4

A healthcare provider, like a genetic counselor or doctor, can counsel relatives and help them get testing.

Why would someone use a Direct Contact Program?

Sharing complex health information about FH with at-risk relatives can be hard.

Talking to a healthcare provider can motivate at-risk relatives to get tested for FH.

If relatives test positive for FH, they can get care for their FH health risk sooner.

Section 3: Direct Contact Program

Please answer the following questions.

You may use the "Back" button at the bottom of the page to return to the information about the Direct Contact Program. Your responses will be saved if you go back to review the information.

* 27. Imagine if your relative agreed to a Direct Contact Program. In this program, your relative gave a healthcare provider the names and contact information of other relatives who are at risk for FH. With your relative’s permission, the healthcare provider contacted family members to tell them that “A family member has received an FH diagnosis and you could be at risk too.”

How helpful would it be if a healthcare provider contacted you or your relatives who may have FH to give information about your relative’s FH diagnosis and explain family members’ risks for heart disease?

- ◯ Very helpful
- ◯ Somewhat helpful
- ◯ Neutral
- ◯ Somewhat unhelpful
- ◯ Not helpful at all
- ◯ Unsure
- ◯ Other, please specify:

* 28. What type of healthcare provider would you or your relatives who may have FH feel comfortable being contacted by about your relative's FH result to talk about the risks for FH and the need to be tested? *(Check all that apply)*

- ☐ Genetic counselor (a healthcare provider with expertise in genetics)
- ☐ Nurse
- ☐ Primary care provider
- ☐ Specialist (e.g., cardiologist, lipidologists, etc.)
- ☐ Pharmacist
- ☐ Medical staff trained for this purpose
- ☐ Other, please specify:

* 29. How acceptable would it be to you or your relatives who may have FH if someone from your relative's healthcare provider's office contacted you or your relatives who may have FH to give information about an FH diagnosis and the risks of heart disease?

- ○ Very Acceptable
- ○ Somewhat Acceptable
- ○ Neutral
- ○ Somewhat Unacceptable
- ○ Very Unacceptable
- ○ Unsure

* 30. If your relative used a Direct Contact program and wanted a healthcare provider to contact you, what would be the best method(s) of reaching you?

- ○ Phone call
- ○ Mail
- ○ Email
- ○ A combination of these methods
- ○ Other, please explain:

IMPACT-FH: Family Member Survey

Section 3: Direct Contact Program (CASCADE)

CASCADE is a new program we are starting with the Direct Contact Program. CASCADE offers genetic counseling, information and support to help individuals consider getting tested for FH. The program also allows family members to order a genetic test kit to their home address, to complete and send back. Family members could choose this option in talking to a provider doing direct contact and/or in a chatbot.

Please read the information below about the CASCADE program and answer the following questions.

"Cascade Testing" for At-Risk Relatives

Geisinger is designing a program, called CASCADE, to help at-risk relatives get cascade testing.

- CASCADE: Contact And Support, Counseling, And DNA Testing Empowerment

What is cascade testing?

- When at-risk relatives get tested for FH, through genetic testing or cholesterol testing, after the first person in a family is diagnosed.

What does CASCADE do?

- Provides genetic counseling for at-risk relatives
- Helps at-risk relatives get genetic testing for FH using a mail-order kit
- Helps at-risk relatives connect with a doctor for cholesterol testing

How much does CASCADE cost?

- After a person receives a positive FH genetic test result, there is a period of time when at-risk relatives can have no-cost genetic testing
- A small fee of $20 will be required for a doctor to order the genetic test
- Testing company works with insurance for testing outside of the no-cost window – self-pay option is $200

* 31. If the CASCADE program was offered to you or family members who may have FH, how would you respond? *Please be detailed in your response.*

* 32. How comfortable would you feel ordering a genetic test kit to your home address after a healthcare provider contacted you about your FH risks?

- Very comfortable
- Somewhat comfortable
- Neutral
- Somewhat uncomfortable
- Totally uncomfortable

IMPACT-FH: Family Member Survey

Section 3: Direct Contact Program

Please answer the following questions.

You may use the "Back" button at the bottom of the page to return to the information about the Direct Contact Program or CASCADE. Your responses will be saved if you go back to review the information.

* 33. Ideally, if a healthcare provider were to contact you or your realtives who may have FH to share information about an FH diagnosis and the risks, what would you want them to say/do?

* 34. How would your other relatives respond if a healthcare provider contacted them to share information about a common relative’s FH diagnosis and their risks?

35. What else do you want to share with us about the Direct Contact Program and/or how to make it better?

IMPACT-FH: Family Member Survey

Additional Opportunities

* 36. Would you be interested in taking part in further opportunities on how healthcare providers can support families talking about FH?

- ◯ Yes
- ◯ No

IMPACT-FH: Family Member Survey

Additional Opportunities - Contact Information

* 37. Please provide your name, phone number and email address so we can contact you with more information on ways to take part in more FH projects:

Name

Email Address

Phone Number

Share a Survey with your Family Members!

We are also interested in understanding how *your family members* feel about these materials.

If you are willing to share this survey with your blood relatives, or your spouse/partner, *please copy the link below and share it with them*.

You may share the link with your family members in any way you choose.

Family Member Survey Link: [Link to Family Member Survey]
